# Genomic subtypes of non-muscle-invasive bladder cancer: guiding immunotherapy decision-making for patients exposed to aristolochic acid

**DOI:** 10.1186/s10020-025-01199-1

**Published:** 2025-04-17

**Authors:** Yun Peng, Yuxuan Song, Caipeng Qin, Mengting Ding, Zixiong Huang, Fei Wang, Yuchao HuangFu, Luping Yu, Yiqing Du, Tao Xu

**Affiliations:** Department of Urology, Peking University People’s Hospital, Beijing, 100044 China

**Keywords:** Non-muscle-invasive bladder cancer, Aristolochic acid, Genomic subtypes, Immunotherapy, Bacillus Calmette-Guerin

## Abstract

**Background:**

The limited genomic data on non-muscle-invasive bladder cancer (NMIBC) hampers our understanding of its carcinogenesis and development. Specifically, Aristolochic acid (AA), a potent human carcinogenic compound from aristolochia plants and commonly found in Chinese herbal medicine, has been extensively documented as being closely associated with the onset and progression of bladder cancer. However, the field of AA-induced NMIBC remains largely unexplored in terms of its genomic and molecular characteristics, as well as clinical therapeutic strategies.

**Methods:**

To bridge this knowledge gap, we conducted a comprehensive study using a cohort of 81 NMIBC samples. We performed whole-exome sequencing (WES) and RNA sequencing (RNA-seq) to obtain detailed genomic and transcriptomic data. We subjected these datasets to genomic analysis and subtype analysis to gain valuable insights into NMIBC.

**Results:**

By temporally dissecting mutations in NMIBC specimens, we identified a comprehensive mutational landscape of NMIBC and the associations of these mutations with recurrence-free survival. Additionally, we discerned four genomic subtypes of NMIBC: AA-like, FGFR3/HRAS, FGFR3 & chr9Del, and genome instability (GI). The AA-like subtype presented a high frequency of gene mutations along with a pronounced AA mutagenesis signature of SBS22 (Fisher test: P-value 3.5e-4, OR 25.25) even after temporal dissection. The FGFR3/HRAS subtype exhibited FGFR3 or HRAS mutations with few copy number alterations (CNAs). The FGFR3 & chr9Del subtype was characterized by the co-occurrence of chr9p and chr9q deletions as well as FGFR3 mutations, while the GI subtype showed a high frequency of CNAs. Notably, the AA-like and GI subtypes demonstrated better outcomes after immunotherapy, whereas the FGFR3/HRAS subtype showed poorer outcomes.

**Conclusions:**

Our findings provide novel perspectives on the genomics of NMIBC, unveiling four prominent genomic subtypes, each showing different outcomes following immunotherapy.

*Trial registration*: No. 2019PHB268-01 (retrospectively registered on February 14, 2020).

**Supplementary Information:**

The online version contains supplementary material available at 10.1186/s10020-025-01199-1.

## Background

Bladder cancer (BCa), accounting for approximately 614,000 new cases and 220,000 deaths annually worldwide, is predominantly composed of non-muscle-invasive bladder cancer (NMIBC), constituting approximately 75% of cases, with the remaining classified as muscle-invasive bladder cancer (MIBC) (Babjuk et al. 2022; Bray et al. 2022). However, due to the relatively better prognosis of NMIBC, coupled with the challenging nature of obtaining its specimens, the scope of genomic research dedicated to NMIBC is rather limited, in contrast to the extensive genomic analyses conducted on MIBC (Gui et al. 2011; Weinstein et al. 2014; Robertson et al. 2017; Hurst et al. 2017).

The standard treatment approach for NMIBC involves resection followed by intravesical infusion chemotherapy or immunotherapy with Bacillus Calmette-Guerin (BCG). Notably, research shows that NMIBC is substantially heterogeneous and that a considerable subset of NMIBCs exhibit an elevated susceptibility to relapse or progress to MIBC or metastatic disease, which are more ominous disease stages. As a result, the prognosis of NMIBC is highly variable and unpredictable (Kulkarni et al. 2010). Furthermore, while patients with NMIBC generally have a longer life expectancy than those with MIBC, elevated rates of local recurrence have been reported, requiring regular cystoscopies (Pilala et al. 2024). These procedures not only compromise patients’ quality of life but also impose a significant financial burden on healthcare systems, making NMIBC one of the most economically challenging forms of cancer (Pilala et al. 2024; James and Gore 2013). Consequently, there is an urgent clinical need for improved prognostication and personalized therapeutics, as well as a pressing need to comprehensively characterize the heterogeneity of NMIBC.

Exposure to aristolochic acid (AA), a potent human carcinogenic compound from *Aristolochia* plants, is known to be closely associated with the onset and progression of BCa (Kang et al. 2021). Our prior research showed that AA can induce clonal expansion and often contribute to the multifocality of BCa (Li et al. 2020). Nevertheless, relatively little attention has been devoted to elucidating its prognostic implications in clinical contexts; additionally, the role of AA in NMIBC, particularly concerning subtypes of NMIBC and therapeutic strategies for NMIBC, remains largely uncharted (Yang et al. 2019). In this study, we assessed the genomic profiles of 81 NMIBC patients and identified four NMIBC subtypes distinguished by unique genomic characteristics and outcomes after immunotherapy. Our findings will inform the design of personalized treatment approaches according to the distinct genomic profiles of individual NMIBC patients.

## Methods

The STROBE reporting guideline was followed for descriptive analysis.

### Study design and data sources

A total of 81 tumor biospecimens and matched blood samples were collected from patients diagnosed with NMIBC, as confirmed by pathological examination. All the samples and clinical information were collected according to protocols approved by the ethics committee of the Peking University People’s Hospital (No. 2019PHB268-01). The clinical and pathological characteristics are summarized in Table S1. DNA and RNA (3 samples without enough qualified RNA) were extracted. Whole-exome sequencing (WES) and RNA-sequencing (RNA-seq) were used to define genomic and transcriptomic characteristics. Somatic mutations were called based on the GATK best practices workflow (DePristo et al. 2011; McKenna et al. 2010). Copy number analysis was performed using alleleCount and ASCAT (v3.1.2) (Van Loo et al. 2021). Expression levels of transcripts were estimated by salmon (Patro et al. 2017) and summarized to the gene level with tximport (Soneson et al. 2016) (For details, see the supplementary methods).

### Classification of driver alterations in NMIBC

Any nonsynonymous variant underwent categorization based on the following criteria. If the mutation was found to be deleterious (either a stop-gain or predicted deleterious mutation according to two out of the three computational approaches applied, namely, Sift (Kumar et al. 2009), Polyphen (Adzhubei et al. 2013) and MutationTaster (Schwarz et al. 2010)) and the gene was annotated as a tumor suppressor in COSMIC (Forbes et al. 2011) and OncoKB (Chakravarty et al. 2017), the variant was classified as a driver mutation. Additionally, if the gene was annotated as an oncogene by COSMIC and we identified ≥ 3 exact matches of the specific variant in COSMIC or if it was labeled oncogenic in OncoKB, we classified the mutation as a driver mutation (Robertson et al. 2017; Jamal-Hanjani et al. 2017; Frankell et al. 2023). In total, we curated a collection of 1366 driver mutations in 502 cancer driver genes (CDGs) for NMIBC (Table S2).

### Temporal dissection of mutations

Several studies have already underscored this complexity in malignancies in which tumors often undergo branching evolution with respect to additional mutations after their initial clonal advantage (Kent and Green 2017 Apr; Tarabichi et al. 2021). Cancers evolve from a single cell through somatic mutations, some of which enable hallmark traits. The descendants of this cell form the initial cancer clone, and over time, selection, mutation, drift, and spatial separation give rise to genetically distinct subpopulations (Burrell et al. 2013; Yates and Campbell 2012). The same procedure was employed as previously described (Jamal-Hanjani et al. 2017; Frankell et al. 2023; McGranahan et al. 2015) to construct the temporal sequence of mutations. The cancer cell fraction (CCF) of each mutation was estimated by considering both tumor purity and local copy number. Mutations were classified as clonal (present in all tumor cells of a sample) if the 95% confidence interval (CI) for the CCF overlapped with 1; otherwise, they were classified as subclonal (present in only a subset of tumor cells in the sample). Clonal mutations were further categorized as “early” or “late” to correct for subclonal copy number alterations (CNAs), as subclonal copy number events may lead to loss of this mutation in a subset of cancer cells. Specifically, mutations in genomic regions with at least two copies of the major allele were initially classified as early if the mutation copy number exceeded 1, and as late if it was less than or equal to 1. In summary, all early clonal events were classified as “early” while late clonal and subclonal events were grouped as “late”. Additionally, we assessed the early enrichment of a CDG by comparing its early mutation ratio with the background early mutation ratios obtained through permutation testing (McGranahan et al. 2015).

### Cancer pathways

For pathway-level analysis, we first obtained 10 oncogenic signaling pathways defined by TCGA (Sanchez-Vega et al. 2018). Additionally, we curated 6 pathways used in other urothelial cancer studies, namely, the histone modification pathway (EP300, CREBBP, KMT2 C/D, KDM6 A, BAP1, ASXL1/2 and SETD2), SWI/SNF pathway (ARID1 A, ARID1B and ARID2), DNA damage pathway (ERCC2, BRIP1, ATM, BRCA1/2, RAD21/50 and CHEK1), Cohesin complex pathway (STAG1/2, RAD21 and SMC1 A/3), oxidative stress pathway (NFE2L2, KEAP1, CUL3 and TXNIP), and alternative splicing pathway (RMB10, SF3B1, U2 AF1 and CDK12) (Robertson et al. 2017).

### Whole-genome doubling (wGD) detection

To accurately estimate wGD events, we utilized two methods and considered samples to have wGD only if both methods detected wGD. The first method, as described in previous publications (Frankell et al. 2023), considered a sample to have wGD if the major allele had a copy number of at least 2 across at least 50% of the genome. The second method determines wGD by representing each sample as an aberration profile of major and minor allele copy numbers at the chromosome arm level and estimating the probabilities of aberration in the chromosome arm (Dewhurst et al. 2014). Then, wGD was estimated according to ploidy and probability data. The two methods showed consistent results for identifying wGD events.

### Definition of hypermutation

Tumor mutational burden (TMB) was first calculated for each sample (Table S1). We reasoned that hypermutated samples would show a higher mutational burden compared to non-hypermutated samples. To define hypermutation, we ranked the samples based on TMB in descending order and then calculated the degree of variability, defined as the difference in TMB with the next sample, as previously reported (Fujii et al. 2021). A significant change in TMB was observed at a specific threshold, indicated by the dashed line in Figure S1. This threshold was selected as the cutoff to distinguish between hypermutated and non-hypermutated samples.

### Estimation of the weighted genome instability index (wGII) and chromosomal instability (CIN)

The wGII was calculated as the proportion of the genome with an aberrant copy number compared to the ploidy. To distinguish CIN, a threshold of 0.2 was used (Burrell et al. 2013). Samples with a wGII value above this threshold were classified as exhibiting CIN, indicating a higher degree of genome instability and aberrant CNAs across the genome.

### Quantitation of mutagenesis by APOBEC cytidine deaminases

We quantified APOBEC cytidine deaminases by considering two specific mutations (C to T and C to G) occurring in the tCw motif (w = A or T) (Robertson et al. 2017). On a per-sample basis, we calculated the enrichment of the APOBEC mutation signature by comparing the fraction of APOBEC-mutated cytosines to the fraction of cytosines occurring in the tCw motif within the upstream and downstream regions of 20 nucleotides surrounding each mutated cytosine. This allowed us to define the APOBEC group based on the APOBEC mutation load (Robertson et al. 2017).

### De novo extraction of mutational signatures

A hierarchical Dirichlet process (HDP) model with priors (commonly active in BCa, including SBS2, SBS5, SBS13, and SBS22) was used to identify the mutagenesis signatures (Frankell et al. 2023; Teh et al. 2006). Subsequently, the expectation maximization (EM) algorithm was employed to deconstruct the newly identified signatures into known etiology signatures reported in COSMIC (v.3.3) (Frankell et al. 2023; Teh et al. 2006). The same analysis framework was also applied to early and late mutations separately.

### Gene-level and pathway-level estimates of selection using dN/dS

Positive mutation selection was estimated using dN/dS, following the methodology outlined in a previous study (Frankell et al. 2023). The dN/dS estimate for point mutations was calculated by combining the dN/dS estimates for missense, nonsense, and splice-site substitutions. The dN/dS timing odds ratio (OR) for each gene was computed as the dN/dS estimate for early mutations divided by the dN/dS estimate for late mutations. Genes with an OR > 2 were classified as early favored, while genes with an OR < 0.5 were classified as late favored. Genes with an OR between 0.5 and 2 were classified as “none.” The results were plotted for all genes with q-values < 0.1 in either early or late mutations (Frankell et al. 2023). Pathways were also classified using a dN/dS OR as described for genes (Frankell et al. 2023).

### Defining mutually exclusive and co-occurring relationships

To identify significantly mutually exclusive and co-occurring relationships between important events, DISCOVER (Frankell et al. 2023) was employed. DISCOVER takes into account the overall distribution of events and allows for a more accurate assessment of the relationships. All mutations, focal and broad CNAs, and mutagenesis signatures along with wGD, hypermutation, CIN, and APOBEC events were considered together to establish an appropriate background. The analysis was focused on events that occurred in at least 10% of tumors (for mutations, only genes in the driver alteration list were considered) in each case. False discovery rate (FDR) correction was applied to account for multiple testing. Additionally, DISCOVER was run separately on early and late events to evaluate their relationships independently.

### Significantly mutated genes (SMGs) and recurrent CNAs

Genes that exhibited a significant excess of nonsynonymous mutations compared to the estimated background mutation density were identified using MutSigCV (v1.4) (Lawrence et al. 2013). Since MutSigCV only provides the hg19 reference genome, the mutations were first converted to the hg19 genome coordinates using the rtracklayer package before running MutSigCV. Additionally, genes that showed significant positive selection were estimated using dndscv (Martincorena et al. 2017). A q-value threshold of less than 0.1 was considered significant. Only CDGs that demonstrated significance in both MutSigCV and dndscv analyses were classified as SMGs.

Recurrent focal and broad CNAs were identified using GISTIC2 (Mermel et al. 2011). GISTIC2 analysis was performed with an amplification and deletion threshold of 0.25 and a broad length cutoff of 0.75. A q-value threshold of less than 0.1 was used as the cutoff to determine significant recurrent CNAs.

### Unsupervised clustering of mutations and CNAs

To define robust groups of samples based on mutations and CNAs, we applied nonnegative matrix factorization (NMF) as previously reported (Weinstein et al. 2014). We first created a binary matrix of genetic events in 81 samples, which comprised 76 frequently observed (occurring in 10% of samples) events and 18 significant focal and 10 broad CNAs. The NMF method with the"brunet"algorithm was used to perform the factorization.

### Definition of subtype scores

For each subtype, we derived the top 30 differentially expressed genes based on the adjusted P value from DESeq2 (Love et al 2014). Then, the 30 genes were grouped by subtype-up and subtype-down based on the direction of log2 FoldChange. GSVA (Hänzelmann et al. 2013) was employed to calculate the gene set scores in the UROMOL (Lindskrog et al. 2021) dataset. Finally, the subtype score, reflecting the overall activity of the subtype, was calculated as the difference between the subtype-up and subtype-down gene set scores.

### Integrative pathway analysis

We assessed somatic mutations and copy number changes at the gene level within the context of cancer pathways. For our analysis, we only considered altered genes with gene mutations in our driver alteration list or CNAs in terms of amplifications and deep deletions determined by GISTIC2 (Mermel et al. 2011). These alterations were assessed within the context of cancer pathways to gain insights into their functional implications in NMIBC.

### Statistical analysis

Statistical analyses were performed using R (R Core Team 2020), version 4.3.1. All P-values were calculated according to a two-sided analysis unless otherwise specified. The Wilcoxon rank-sum test was used for group comparisons. The survival (Therneau 2015) package was utilized for univariate and multivariate cox regression analyses. The rms (Harrell 2015) package was employed to calculate variance inflation factors (VIF) (Miles 2014), and only multivariable cox regression models with all variables having VIF < 5 were used. Decision curve analysis (DCA) (Vickers and Elkin 2006) was conducted using the dcurves (Sjoberg et al. 2024) package. The Benjamini-Hochberg (BH) correction method was applied to adjust the P-values.

## Results

### Clinical and pathological characteristics

A total of 81 tumor biospecimens and matched blood samples were collected from patients diagnosed with NMIBC as confirmed by pathological examination. The cohort comprised 58 stage Ta, 22 stage T1, and 1 stage Tis cases. All patients underwent transurethral resection of bladder tumor (TURBT) and had not received any previous intravesical therapy, chemotherapy, or radiotherapy. Tumor grades included 38 high-grade (HG), 32 low-grade (LG), and 11 papillary urothelial neoplasm of low malignant potential (PUNLMP). The majority of patients (56 out of 81, accounting for 69.1%) received intravesical chemotherapy following surgery, while 14 patients received BCG treatment. The median follow-up period was 22 months (ranging from 4 to 73 months). No significant difference in recurrence-free survival (RFS) was observed among patients receiving different intravesical therapies (P-value: 0.91). The clinical and pathological characteristics are summarized in Table S1.

### Somatic mutational landscapes and temporal dissection of mutations

**Fig. 1:**
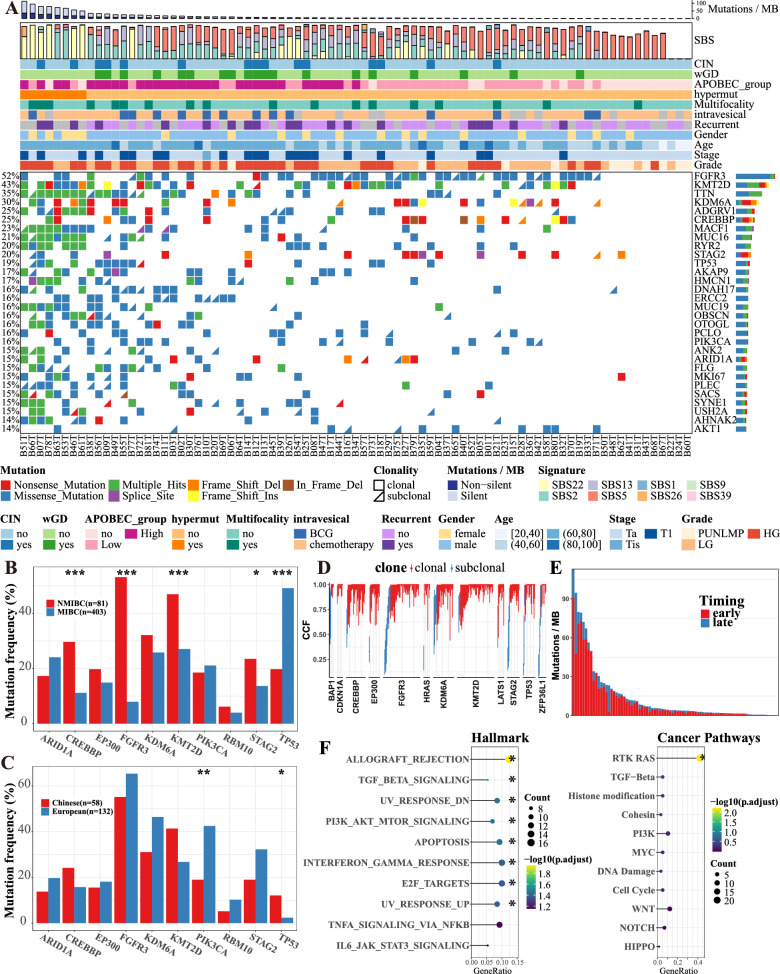
Landscape of genomic mutations in NMIBC. **A** Oncoplot of the top 30 genes in NMIBC. **B** The mutation frequency was compared between our cohort (NMIBC) and TCGA data (MIBC). **C** The mutation frequency was compared between our cohort (Chinese) and other NMIBC cohorts (European). Importantly, this comparison was limited to the Ta stage, as other cohort studies primarily focused on the Ta stage. **D** Mutations were classified as clonal and subclonal based on cancer cell fractions. Only further identified driver genes are depicted. **E** Mutations were then temporally classified as “early” or “late” based on clonality and mutation copy number. **F** Functional enrichment analysis was used to define the pathways of genes with a higher early mutation rate than early background mutation rate. Label (*) means P < 0.05, label (**) means P < 0.01, and label (***) means P < 0.001

Using Mutect2, a total of 68,672 somatic mutations were identified, including 66,309 single-nucleotide variants, 325 multinucleotide variants, and 2038 indels. The nonsynonymous mutation rate ranged from 0.02 to 35.88 per megabase (MB), with a mean of 4.55 and a median of 1.44 (Fig. 1A). Notably, this rate was lower than that observed for MIBC (mean 8.2 and median 5.8) (Robertson et al. 2017). Compared to MIBC, NMIBC exhibited a distinct mutational profile, with an enrichment of mutations in CREBBP (30% versus 11%), FGFR3 (53% versus 8%), KMT2D (47% versus 27%), and STAG2 (23% versus 14%), while showing a lower mutation frequency in TP53 (20% versus 49%) (Fig. 1B) (Robertson et al. 2017). In contrast to European cohorts (Hurst et al. 2017; Balbás-Martínez et al. 2013; Nordentoft et al. 2014), the present Chinese cohort exhibited a greater mutation frequency in TP53 (12% versus 2% in European) but a reduced mutation frequency in PIK3 CA (19% versus 43% in European) among NMIBCs (only NMIBCs of stage Ta were considered) (Fig. 1C and Table 1). Based on the overall mutational burden, a total of 8 samples were classified as hypermutated (Figure S1).

**Table 1:** The Comparative Analysis of the Mutational Landscape in Ta Stage NMIBC: The mutation frequencies of individual genes were compared between European and Chinese cohorts using a Fisher’s exact test. We only considered Ta stage for consistent with other cohorts. An adjusted P-value < 0.1 was considered significant

	Nordentoft et al. (Denmark)	Balbás-Martínez et al. (Spain)	Hurst et al. (UK)	Combined European cohort	Present Chinese cohort	P value	Adj. pval
	n = 20	n = 25	n = 82	n = 127	n = 58		
ARID1A	7 (35%)	3 (12%)	15 (18%)	25 (20%)	8 (14%)	0.410	0.456
CREBBP	4 (20%)	4 (16%)	12 (15%)	20 (16%)	14 (24%)	0.219	0.313
EP300	5 (25%)	3 (12%)	15 (18%)	23 (18%)	9 (16%)	0.834	0.834
FGFR3	8 (40%)	10 (40%)	65 (79%)	83 (65%)	32 (55%)	0.195	0.313
KDM6A	13 (65%)	3 (12%)	43 (52%)	59 (46%)	18 (31%)	0.055	0.150
KMT2D	3 (15%)	6 (24%)	25 (30%)	34 (27%)	24 (41%)	0.060	0.150
PIK3CA	5 (25%)	5 (20%)	44 (54%)	54 (43%)	11 (19%)	1.69e–03	0.017
RBM10	4 (20%)	1 (4%)	8 (10%)	13 (10%)	3 (5%)	0.398	0.456
STAG2	5 (25%)	6 (24%)	30 (37%)	41 (32%)	11 (19%)	0.078	0.156
TP53	1 (5%)	2 (8%)	0 (0%)	3 (2%)	7 (12%)	0.012	0.058

We then temporally classified mutations as early or late, resulting in 55,393 early mutations and 13,227 late mutations (Fig. 1D, E). Overrepresentation analysis revealed that CDGs with a higher early mutation rate than an early background mutation rate were significantly enriched (adjusted P-value < 0.05) in several pathways, including the TGF_BETA_SIGNALING, PI3K_AKT_MTOR_SIGNALING, INTERFERON_GAMMA_RESPONSE, and E2F_TARGETS pathways based on the MSigDB Hallmark Gene Set. Additionally, the RTK-RAS pathway within cancer pathways also exhibited significant enrichment (Fig. 1F).

### Mutation selection and mutagenesis signature

**Fig. 2:**
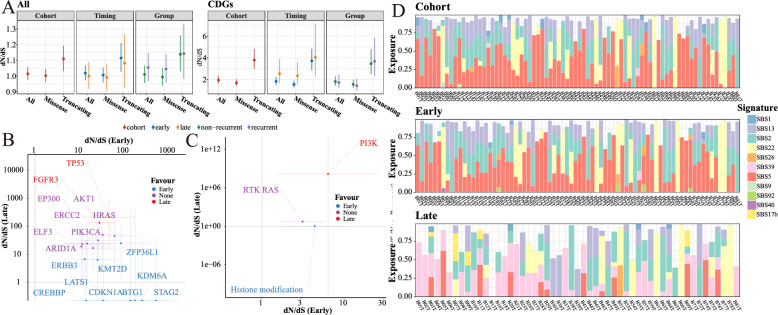
Temporal selection and mutagenesis signature. **A** Comparison of the overall amount of selection in all genes and CDGs. The line ranges represent the 95% CI of the dN/dS ratio. **B**, **C** Gene-level **B** or pathway-level **C** selection of point mutations, measured by comparing the dN/dS ratio of early mutations and late mutations. The error bars indicate the 95% CI. **D** Significant signature exposures. A mutational signature was considered present if it accounted for at least 10 mutations and contributed to at least 5% of the total mutations in a sample

Positive mutation selection was then estimated using dN/dS. When considering the global dN/dS of all genes, significant positive selection was observed for truncating mutations. Moreover, the selection was slightly stronger for early mutations and recurrent samples than for late mutations and nonrecurrent samples (Fig. 2A). Strong positive selection of CDGs was observed for both missense mutations and truncating mutations, although they were temporally dissected (Fig. 2A). These data suggested that positive selection of CDGs plays a significant role in the NMIBC, with evidence of selection observed across both early and late mutations.

By comparing dN/dS between early and late mutations, we were able to quantify the preference for early or late selection in CDGs or pathways (Frankell et al. 2023). Notably, histone modification genes (KMT2D, KDM6 A, and CREBBP) exhibited stronger early selection, whereas FGFR3 and TP53 were under stronger late selection, reinforcing their role in NMIBC progression (Fig. 2B). Consistent with the gene-level analysis, the histone modification pathway showed higher early selection, while the PI3 K pathway demonstrated stronger late selection (Fig. 2C).

The mutational signatures were extracted de novo using the HDP and the expectation maximization algorithm (Figures S2-6) (for details, see Methods) (Frankell et al. 2023). A mutational signature was deemed present if it accounted for at least 10 mutations and contributed to at least 5% of the total mutations of a sample (Fig. 2D). The aging-related mutagenesis signature SBS5 was detected in 70 samples and accounted for approximately 9788 mutations (14.76% of total mutations). On the other hand, the AA-mediated signature SBS22 contributed to the highest number of mutations (25,989 mutations, 39.19% of total mutations), but it was observed only in 22 samples. This indicated that AA exposure often contributed to hypermutated samples (Fig. 1A). Differential expression analysis (Table S3) and GSEA indicated that SBS22 was associated with the activation of Neutrophil extracellular trap formation, HALLMARK_G2M_CHECKPOINT and HALLMARK_E2 F_TARGETS (Figure S7). Two APOBEC-mediated signatures prevalent in MIBC were also observed in NMIBC (SBS2: 55 samples, 16,998 mutations of 25.63%; SBS13: 48 samples, 7130 mutations of 10.75%) (Fig. 2D).

Through the temporal dissection of mutations, we demonstrated the importance of an uncharacterized signature, SBS39 (presented in 39 samples), in late mutations (Fig. 2D), suggesting a potential role in subclonal expansion. To characterize its function in NMIBC, Differential expression analysis (Table S4) and GSEA revealed that late SBS39 was linked to the inactivation of several immune-related pathways, including HALLMARK_INTERFERON_GAMMA_RESPONSE, HALLMARK_INFLAMMATORY_RESPONSE, and HALLMARK_INTERFERON_ALPHA_RESPONSE (Figure S8). Remarkably, APOBEC-mediated mutagenesis signatures were prevalent in both early and late mutations (Fig. 2D). Furthermore, we estimated the mutation load attributed to APOBEC cytidine deaminases and observed a remarkable enrichment of APOBEC-mediated mutations in 62 samples (76.54% of total samples) (Fig. 1A).

### SMGs and recurrent CNAs

**Fig. 3:**
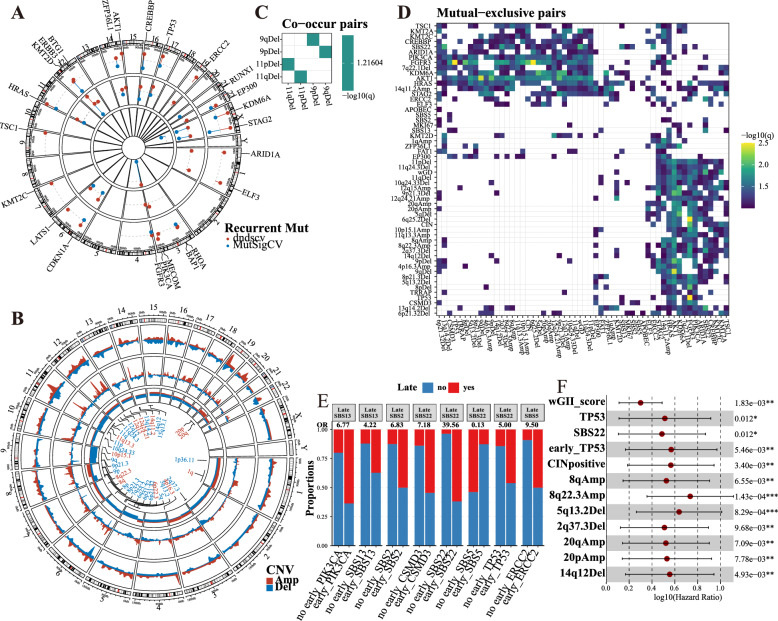
Significant mutated genes (SMGs) and evolutionary dependencies. **A** The -log10 (q value) was represented by the distance from 0-baseline (as indicated by the dotted line) for significant genes (q value < 0.01 in either dndscv or MutSigCV) in all mutations (outermost circular track), early mutations (intermediate circular track), and late mutations (innermost circular track). **B** The genomic density plot displays the distribution of amplified (Amp) and deleted (Del) CNAs across all samples. A higher density signifies that a greater number of samples have CNA in that region (outermost circular track). The two innermost circular tracks depict the G-score and frequency returned by GISTIC2 for focal and broad CNAs, providing additional information on the significance and prevalence of these alterations. Significant recurrent CNAs were labeled. **C**, **D** Co-occurrence (**C**) and mutual exclusivity (**D)** relationships among all genomic events. Only significant pairs are shown (**E**) Ordering interactions can influence the likelihood of a late event occurring based on the presence of early events. An odds ratio (OR) greater than 1 indicates co-occurrence, meaning that the early event increases the probability of the late event. Conversely, an OR less than 1 suggests mutual exclusivity, indicating that the early event reduces the probability of the late event. **F** The forest plot of Cox regression in reference to RFS. The corresponding hazard ratio (HR) and 95% confidence interval (CI) are shown

MutsigCV and dndscv identified 11 SMGs (q value < 0.1 for both algorithms), namely, BAP1, CDKN1 A, CREBBP, EP300, FGFR3, HRAS, KDM6 A, KMT2D, STAG2, TP53 and ZFP36L1 (hypermutated samples were excluded to prevent bias due to their exceptionally large number of mutations) (Fig. 3A). By temporally dissecting mutations, we identified SMGs that may have been overlooked by the inclusion of late mutations or SMGs associated with subclonal expansions (McGranahan et al. 2015). In this way, we identified the significance of LATS1 mutation in early tumor evolution (Fig. 3A). Differential expression analysis (Table S5) and GSEA indicated that the early mutation of LATS1 was associated with the activation of several immune-related pathways, including Neutrophil Extracellular Trap Formation, Cytokine − Cytokine Receptor Interaction, B Cell Receptor Signaling Pathway, HALLMARK_INFLAMMATORY_RESPONSE, and HALLMARK_INTERFERON_GAMMA_RESPONSE (Figure S9). Additionally, FGFR3 mutations were significantly identified in late events, suggesting a potential association with NMIBC subclonal expansions (Fig. 3A). Furthermore, the GISTIC2 tool was utilized to identify 23 significant focal CNAs (8 amplifications and 15 deletions) and 10 arm-level CNAs (4 amplifications and 6 deletions) (Fig. 3B). Similar to MIBC, the most frequently recurrent focal CNAs were observed in the 9p21.3 deletion samples (25 samples, 30.86%) (containing CDKN2 A). In addition, the deletions of 8p21.3 (23 samples, 28.40%) and 6p21.32 (22 samples, 27.16%) were also observed to be significantly recurrent (Fig. 3B).

### Genomic dependency analysis

It is widely recognized that there is a significant context dependency in cancer, particularly between genomic events driven by synthetic lethality or functional redundancy (Frankell et al. 2023). DISCOVER (Frankell et al. 2023; Canisius et al. 2016), which takes into account the overall distribution of events, was employed to define the co-occurring and mutually exclusive relationships between genomic events. Significant co-occurrence was observed between the deletion of 9p and 9q as well as between the deletion of 11p and 11q (Fig. 3C). Additionally, FGFR3, HRAS, PIK3 CA, and KDM6 A, along with SBS22, were mutually exclusive with numerous focal and broad CNAs as well as certain genomic characteristics, such as CIN and wGD (Fig. 3D). Significant context dependency was also found in early events (Figure S10).

We next investigated whether early genomic events were linked to an elevated or reduced probability of subsequent late events. As expected, an increased likelihood of late SBS2, SBS13, and SBS22 was observed following early SBS2 (OR: 6.83, adjusted P-value: 0.03), SBS13 (OR: 4.22, adjusted P-value: 0.099), and SBS22 (OR: 39.56, adjusted P-value: 8.69e-07), respectively. Early ERCC2 mutation was associated with a higher probability of late SBS5. In addition, a mutually exclusive pattern was observed between early SBS5 and late SBS22 (OR = 0.13, adjusted P-value: 0.01) (Fig. 3E).

### Associations with bladder cancer recurrence

Cox regression was further utilized to explore the association with RFS. Interestingly, we noted that the wGII (HR: 2.00, CI 1.29–3.08; P-value: 1.83e-3) and its binary format, CIN (HR: 3.68, CI 1.54–8.79; P-value: 3.4e-3), and numerous focal (including 2q37.3Del, 5q13.2Del, 14q12Del, and 8q22.3 Amp) and broad CNAs (including 8qAmp, 20pAmp, and 20qAmp) were significantly associated with increased recurrence risk (Fig. 3F and Figure S11 A). The same was also observed for both TP53 and SBS22 (Fig. 3F and Figure S11 A). Then, patients were divided into two groups (recurrent and nonrecurrent) based on their recurrence status within a 2-year follow-up period. Notably, the significance of these associations persisted when compared using logistic regression (Figure S11B).

### Identification of genomic subtypes for NMIBC

**Fig. 4:**
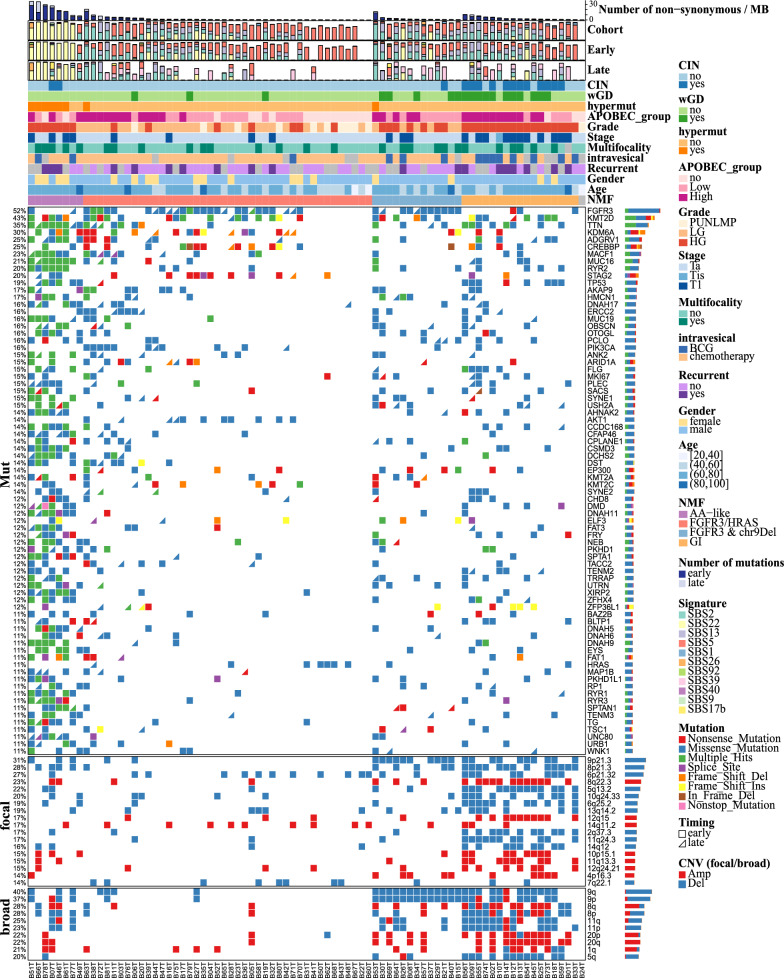
Four genomic subtypes were identified using NMF. Oncoplot of genomic events used to define the genomic subtypes. CIN: chromosomal instability, wGD: whole-genome doubling

**Fig. 5:**
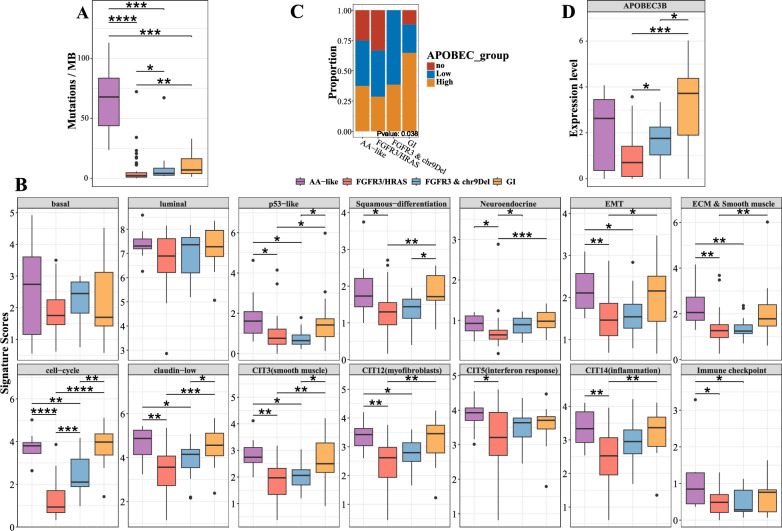
Characteristics of the genomic subtypes. **A** Comparison of the number of mutations per MB across genomic subtypes using the Wilcoxon rank-sum test. **B** Gene expression signature scores of canonical gene sets of BCa, compared between different genomic subtypes. **C** Distribution of the APOBEC group across the genomic subtypes, with p-values calculated using Fisher’s test. **D** Boxplot of APOBEC3B expression, compared between different genomic subtypes using the Wilcoxon rank-sum test. Label (*) means P < 0.05, label (**) means P < 0.01, and label (***) means P < 0.001

NMF analysis identified four distinct genomic subtypes (Fig. 4, Figure S12) according to genomic characteristic profiling of NMIBC. Interestingly, the AA-like subtype presented a high frequency of gene mutations along with a high mutagenesis signature of SBS22 (Fisher test: P value 3.5e-4, OR 25.25) even after temporal dissection (early: P value 2.4e-4, OR 27.43; late: P value 2.22e-07, OR Infinite). The FGFR3/HRAS subtype was defined by the presence of either FGFR3 mutation or HRAS mutation, with fewer focal or broad CNAs. In addition, the FGFR3 & chr9Del subtype was characterized by the co-occurrence of deletions in both 9p and 9q, accompanied by FGFR3 mutation, while the genome instability (GI) subtype was characterized by a high frequency of CNAs and enriched with wGD (Fisher test: P value 4.45e-05, OR 12.01) and CIN (Fisher test: P value: 3.77e-08, OR: 43.33) (Fig. 4). Intriguingly, we observed an apparent increase in tumor mutational burden across the FGFR3/HRAS, FGFR3 & chr9Del, GI, and AA-like subtypes (Fig. 5A).

To comprehensively characterize the subtypes, we then compared the expression levels of some canonical markers of BCa (Fig. 5B and Figure S13 A) (For method details, see the supplementary methods). No differences between basal or luminal scores were observed across the four subtypes, while the expression of luminal markers was higher than that of basal markers (Fig. 5B), indicating that NMIBC is predominantly of the luminal subtype compared with MIBC, which manifests both luminal and basal subtypes (Robertson et al. 2017). We further validated this by defining the transcriptional subtype and identified 5 genomically unstable (GU) samples, along with 73 urothelial-like (Uro) samples (including 7 UroB and 66 UroA) which corresponded to the luminal subtypes (Cotillas et al. 2024) (Table S1). Intriguingly, p53 markers, stroma-related markers, inflammation markers and claudin-low markers were all higher in AA-like and GI subtypes than in the FGFR3/HRAS or FGFR3 & chr9Del subtypes (Fig. 5B). Moreover, immune checkpoint and interferon response scores were also elevated in the AA-like subtype (Fig. 5B). Differential expression analysis (Table S6-9) and GSEA revealed that the HALLMARK_INFLAMMATORY_RESPONSE pathway was upregulated in the AA-like subtype and downregulated in the FGFR3/HRAS subtype (Figure S13B).

Given the significance of APOBEC in BCa (Robertson et al. 2017; Hurst et al. 2017), we further investigated the APOBEC-related mutation load across the genomic subtypes and found significant difference (Fig. 5C). This prompted us to compare the expression of APOBEC family members, leading to the discovery that APOBEC3B was gradually elevated in the FGFR3/HRAS, FGFR3 & ch9Del and GI subtypes (Fig. 5D and Figure S13 C). Differential expression analysis (Table S10) and GSEA indicated that APOBEC mutation load was associated with the activation of the Cell Cycle and HALLMARK_E2 F_TARGETS pathways in samples from the FGFR3/HRAS, FGFR3 & chr9Del, and GI subtypes (Figure S13D).

### Genomic subtypes guiding immunotherapy decisions for NMIBC

**Fig. 6:**
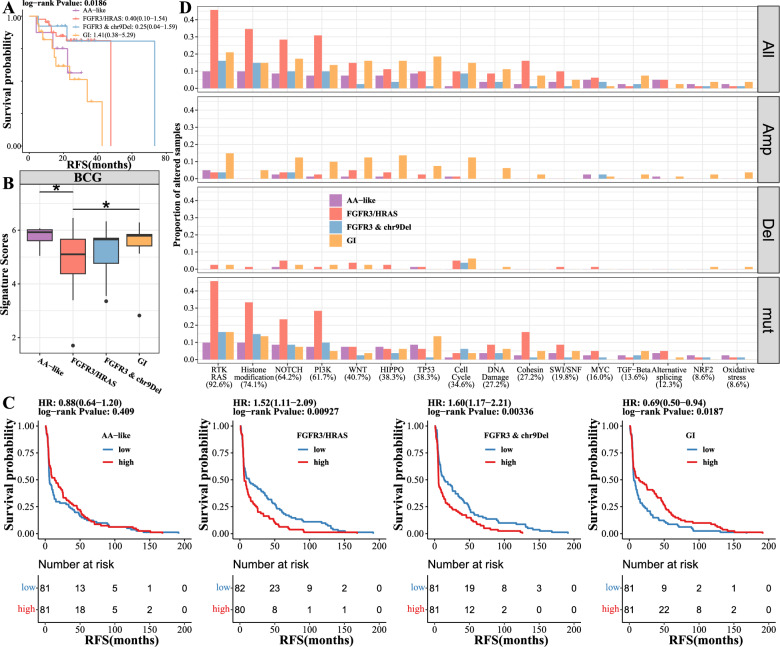
Genomic subtypes guiding immunotherapy decisions for NMIBC. **A** Kaplan‒Meier plot across different genomic subtypes was depicted. **B** Comparison of BCG response scores between genomic subtypes. Label (*) means P < 0.05, label (**) means P < 0.01, and label (***) means P < 0.001. The hazard ratio (HR) with its corresponding 95% CI is indicated, with reference to the AA-like subtype. **C** The low and high groups were defined based on the median subtype score, and Kaplan‒Meier analysis was used to compare the survival outcomes between the two groups. **C** Altered pathways across different genomic subtypes were examined

Consistent with the prognosis finding in genomic events (Fig. 3F), the AA-like and GI subtypes exhibited the poorest survival outcomes in terms of RFS (Fig. 6A). We then assessed the relationships between genomic subtypes and clinical characteristics and found that genomic subtypes were significantly associated with T stage, tumor grade, and EORTC scores, with a gradual escalation observed among the FGFR3/HRAS, FGFR3 & chr9Del, GI, and AA-like subtypes (Figure S14 A). After adjusting for clinical characteristics, we reassessed the associations between genomic subtypes and RFS using multivariable Cox regression models and found no significant associations (Figure S14B). Notably, the genomic subtypes performed similarly to stage and EORTC scores in terms of prognostic prediction (Figure S14 C).

Subgroup analysis suggested a trend toward improved outcomes in the FGFR3/HRAS and FGFR3 & chr9Del subtypes following intravesical chemotherapy (Figure S14D), which persisted after adjusting for clinical characteristics (Figure S14E). We then compared the RFS of the BCG-treated groups and found that the AA-like and GI subtypes tended to exhibit better outcomes, though no significant difference was observed (Figure S14 F). Unfortunately, we were unable to adjust for clinical characteristics in the BCG-treated groups due to small sample sizes. To validate our findings, we compared the expression levels of the BCG immunotherapy response signature (Kim et al. 2010) and found significantly higher scores (Fig. 6B) in the AA-like and GI subtypes. For further validation, we compared our subtype scores in the UROMOL (Lindskrog et al. 2021) dataset, in which 163 patients were treated with BCG. Log-rank analysis showed that the FGFR3/HRAS (HR: 1.52, CI 1.11−2.09, P-value: 0.00927) and FGFR3 & chr9Del (HR: 1.60, CI 1.17−2.21, P-value: 0.00336) subtypes were significantly associated with poor outcomes, while the GI subtype (HR: 0.69, CI 0.50−0.94, P-value: 0.0187) was significantly associated with a preferable outcome (Fig. 6C). The AA-like subtype (HR 0.88, CI 0.64−1.20, P-value: 0.409) also showed a trend toward a preferable outcome, though without statistical significance (Fig. 6C).

We then conducted a comprehensive analysis of somatic mutations and CNAs at the gene level to assess the frequency of pathway alterations in NMIBC. Our findings revealed that the RTK-Ras pathway was altered in 92.6% of the samples, indicating its high relevance in the disease. Furthermore, histone modification, a crucial process involved in epigenetic regulation, was altered in 74.1% of all samples (Fig. 6D). Interestingly, these findings differed from those observed for MIBC, where alterations in the TP53/cell cycle pathway were dominant. This suggests that distinct molecular mechanisms may underlie the development and progression of NMIBC and MIBC.

## Discussion

NMIBC, the most common type of BCa, poses a significant clinical challenge due to a notable propensity for local recurrence though a relatively favorable life expectancy (Babjuk et al. 2022). Here, we evaluated the genomic and transcriptional profiles of 81 NMIBCs to unravel the temporal dynamics of the disease, with the ultimate goal of identifying key features that can guide decision-making regarding therapeutic interventions.

By temporally dissecting mutations, we emphasized the significance of two cancer pathways, RTK-RAS and histone modification (especially KMT2D, KDM6 A and CREBBP), in the initiation of NMIBC due to the high enrichment in early mutations and high early selection preference, respectively. The oncogenic potential of activated RTK/RAS components in BCa has been extensively documented (Wu et al. 2015; Wu 2005). Histone modification has been suggested to define subtypes of MIBC (Meghani et al. 2022; Vos et al. 2020) has been identified in NMIBC (Hurst and Knowles 2022), with prior studies indicating its potential as a therapeutic target in BCa (Zhang et al. 2024). Our findings enhance this knowledge by highlighting the temporal relevance of histone modification in the early development of NMIBC. Additionally, gene-level analysis highlighted more significance in the late selection of FGFR3 and TP53, two common tumorigenic genes of BCa (Ascione et al. 2023). This finding suggested that FGFR3 and TP53 may contribute to the progression and recurrence of NMIBC, as indicated by their stronger late selection in our cohort.

Additionally, temporal analysis of SMGs identified LATS1 as a significant gene in early NMIBC evolution, consistent with its known role as a tumor suppressor in the hippo signaling pathway in BCa. Notably, mutations of LATS1 has been observed in BCa and found to be associated with the BCa response to immune checkpoint inhibitor therapy (Li et al. 2022; Saadeldin et al. 2015). The temporal mutagenesis signature revealed the importance of SBS39 in the subclonal expansion of NMIBC, while SBS39 has been identified to be enriched in cancer mutation hotspots (Wong et al. 2022) but reported sparingly in BCa. Collectively, these findings deepen our understanding of NMIBC development and highlight potential biomarkers, such as LATS1, for further investigation in NMIBC.

The limited representation of diverse racial and ethnic groups among patient populations has impeded our comprehension of potential variations in subtype classification of NMIBC. In addition, efforts to subdivide NMIBC based on genomic features have been confined to specific tumor stages, thus limiting their broader applications (Hurst et al. 2017, 2012). Here, we contributed to addressing this gap by providing comprehensive genomic and transcriptome profiles of a Chinese population, notably characterized by elevated AA exposure (Yang et al. 2014), and the comprehensive genomic and prognostic data enabled us to identify genomic subtypes across all NMIBC stages. In this study, we identified four distinct subtypes by characterizing the genomic profiles of NMIBC. Compared with genomic subtypes (GS1 and GS2 or Clusters 1–3 in Ta) defined by Hurst (Hurst et al. 2017, 2012), GS1 or Cluster 1 and Cluster 2a, characterized by FGFR3 mutation and few CNAs, correspond to FGFR3/HRAS. GS2 or Cluster 2b and Cluster 3, characterized by a high frequency of chr9Del and FGFR3 mutation, correspond to FGFR3 & chr9Del. Hurst (Hurst et al. 2012) also defined 4 main groups (Clusters 1–4) for stage T1 NMIBC. Cluster 1, characterized by chr9Del and FGFR3 mutation aligns with FGFR3 & chr9Del, and Clusters 2–4, characterized by chromosomal instability, would be classified as the GI subtype. The three subtypes (FGFR3/HRAS, FGFR3 & chr9Del, GI) showed differential expression of APOBEC3B, suggesting that APOBEC mutagenesis may shape the three subtypes. Additionally, we also revealed a novel subtype (AA-like) characterized by AA exposure (SBS22) and an extensive mutation load. Furthermore, we provided evidence supporting the prognostic relevance of our subtypes in the UROMOL dataset, underscoring the potential clinical utility of this genomic classification for NMIBC.

Given the growing emphasis on precision medicine, integrating genomic data into clinical practice has the potential to enable more personalized therapeutic approaches, ultimately improving patient outcomes (Olislagers et al. 2025). Risk stratification plays a critical role in tailoring treatment plans. By identifying high-risk patients early, clinicians can prioritize more aggressive therapies. Additionally, high-risk patients may require more intensive surveillance to detect recurrence or metastasis at an early stage (Babjuk et al. 2022). The genomic subtypes showed performance similar to tumor stage and EORTC scores in predicting recurrence in our cohort, suggesting they may have potential as indicators for risk stratification, pending further clinical validation. Importantly, the AA-like and GI subtypes demonstrated higher expression of p53 markers, claudin-low markers, stroma-related markers and BCG-response-related markers, indicating their resistance to chemotherapy and the potential advantages of treating these NMIBC subtypes with immunotherapy (Kim et al. 2010; Dadhania et al. 2016; Choi et al. 2014). Subgroup analysis showed that the AA-like and GI subtypes had better outcomes following BCG therapy, and further validation in the UROMOL dataset confirmed these findings. This observation may be attributed to the increased mutational burden in these subtypes (Fig. 5C), which could lead to the generation of neoantigens recognized by the immune system (Xie et al. 2023). Therefore, although AA-like and GI subtypes are associated with poorer RFS, they may be more immunogenic, making them suitable candidates for immunotherapy. However, prospective trials are needed to determine their actual therapeutic benefit. Given that BCG treatment failure is a major risk factor for disease progression and death in BCa, identifying patients who do not benefit from treatment is critical, as is exploring alternatives to radical surgery (Kamat et al. 2018; Zuiverloon et al. 2012). These findings highlight the potential for clinical implications of the genomic subtypes.

In conclusion, we have provided new insights into the genomics of NMIBC by identifying four major genomic subtypes. Our findings enhance the understanding of the dynamic processes underlying NMIBC and hold the potential to inform clinical decision-making and treatment strategies. While our study offers valuable insights, several limitations must be acknowledged. Our dataset, derived from a single institution and a modest sample size (n = 81), may limit the generalizability of our findings, including the prognostic significance of the identified subtypes. The focus on a Chinese cohort with elevated AA exposure may not fully reflect NMIBC in other populations, necessitating multi-center studies for broader validation. Bulk sequencing may overlook intratumor heterogeneity, particularly in multifocal tumors, potentially affecting the robustness of our subtype classifications and temporal mutation analyses. Single-cell sequencing could refine our understanding of clonal dynamics. Additionally, the small number of BCG-treated patients (n = 14) in our cohort limits the statistical power to confirm immunotherapy responses, highlighting the need for larger, prospective studies to substantiate these preliminary observations.

## Supplementary Information

Additional file 1

Additional file 2

Additional file 3

Additional file 4

Additional file 5

Additional file 6

Additional file 7

Additional file 8

Additional file 9

## Data Availability

The human sequence data generated in this study are not publicly available due to patient privacy requirements but are available upon reasonable request from the corresponding author. Other data generated in this study are available within the article and its supplementary data files. Additionally, the code used in our study has been deposited in the following GitHub repository: https://github.com/Yunuuuu/biomisc/tree/nmibc_subtypes.

## Abbreviations

BCa: Bladder cancer
MIBC: Muscle invasive bladder cancer
NMIBC: Non-muscle invasive bladder cancer
BCG: Bacillus Calmette-Guerin
TURBT: Transurethral resection of bladder tumor
PUNLMP: Papillary urothelial neoplasm of low malignant potential
LG: Low-grade
HG: High-grade
RFS: Recurrence free survival
WES: Whole-exome sequencing
RNA-seq: RNA sequencing
CCF: Cancer cell fraction
CI: Confidence interval
wGD: Whole genome doubling
wGII: Eighted genome instability index
CIN: Chromosomal instability
AA: Aristolochic acids
GU: Genomically unstable
GI: Genome instability
CNA: Copy number alteration
PD: Progressive disease
SD: Stable disease
PR: Partial response
CR: Complete response
CIT: Cartes d’identité des tumeurs
ECM: Extracellular matrix
CDG: Cancer driver gene

## References

[CR1] Adzhubei I, Jordan DM, Sunyaev SR. Predicting functional effect of human missense mutations using PolyPhen-2. Curr Protocols Human Genetics. 2013. 10.1002/0471142905.hg0720s76. 10.1002/0471142905.hg0720s76 PMC4480630 23315928

[CR2] Ascione CM, Napolitano F, Esposito D, Servetto A, Belli S, Santaniello A, et al. Role of FGFR3 in bladder cancer: Treatment landscape and future challenges. Cancer Treat Rev. 2023. 10.1016/j.ctrv.2023.102530. 36898352 10.1016/j.ctrv.2023.102530

[CR3] Babjuk M, Burger M, Capoun O, Cohen D, Compérat EM, Dominguez Escrig JL, et al. European association of urology guidelines on non–muscle-invasive bladder cancer (Ta, T1, and Carcinoma in Situ). Eur Urol. 2022;81(1):75–94. 34511303 10.1016/j.eururo.2021.08.010

[CR4] Balbás-Martínez C, Sagrera A, Carrillo-de-Santa-Pau E, Earl J, Márquez M, Vazquez M, et al. Recurrent inactivation of STAG2 in bladder cancer is not associated with aneuploidy. Nat Genet. 2013;45(12):1464–9. 24121791 10.1038/ng.2799 PMC3840052

[CR5] Bray F, Laversanne M, Sung H, Ferlay J, Siegel RL, Soerjomataram I, et al. Global cancer statistics 2022: GLOBOCAN estimates of incidence and mortality worldwide for 36 cancers in 185 countries. CA: A Cancer J Clin. 2024;74(3):229–63. 10.3322/caac.21834 38572751

[CR6] Burrell RA, McGranahan N, Bartek J, Swanton C. The causes and consequences of genetic heterogeneity in cancer evolution. Nature. 2013;501(7467):338–45. 24048066 10.1038/nature12625

[CR7] Burrell RA, McClelland SE, Endesfelder D, Groth P, Weller MC, Shaikh N, et al. Replication stress links structural and numerical cancer chromosomal instability. Nature. 2013;494(7438):492–6. 23446422 10.1038/nature11935 PMC4636055

[CR8] Canisius S, Martens JWM, Wessels LFA. A novel independence test for somatic alterations in cancer shows that biology drives mutual exclusivity but chance explains most co-occurrence. Genome Biol. 2016;17(1):261. 27986087 10.1186/s13059-016-1114-x PMC5162102

[CR9] Chakravarty D, Gao J, Phillips S, Kundra R, Zhang H, Wang J, et al. OncoKB: a precision oncology knowledge base. Jco Precis Oncol. 2017;1:1–16. 10.1200/PO.17.00011 PMC5586540 28890946

[CR10] Choi W, Porten S, Kim S, Willis D, Plimack ER, Hoffman-Censits J, et al. Identification of distinct basal and luminal subtypes of muscle-invasive bladder cancer with different sensitivities to frontline chemotherapy. Cancer Cell. 2014;25(2):152–65. 24525232 10.1016/j.ccr.2014.01.009 PMC4011497

[CR11] Cotillas EA, Bernardo C, Veerla S, Liedberg F, Sjödahl G, Eriksson P. A versatile and upgraded version of the lundtax classification algorithm applied to independent cohorts. J Mol Diagn. 2024;26(12):1081–101. 39326668 10.1016/j.jmoldx.2024.08.005

[CR12] Dadhania V, Zhang M, Zhang L, Bondaruk J, Majewski T, Siefker-Radtke A, et al. Meta-analysis of the luminal and basal subtypes of bladder cancer and the identification of signature immunohistochemical markers for clinical use. EBioMedicine. 2016;1(12):105–17. 10.1016/j.ebiom.2016.08.036 PMC5078592 27612592

[CR13] DePristo MA, Banks E, Poplin R, Garimella KV, Maguire JR, Hartl C, et al. A framework for variation discovery and genotyping using next-generation DNA sequencing data. Nat Genet. 2011;43(5):491–8. 21478889 10.1038/ng.806 PMC3083463

[CR14] Dewhurst SM, McGranahan N, Burrell RA, Rowan AJ, Grönroos E, Endesfelder D, et al. Tolerance of whole-genome doubling propagates chromosomal instability and accelerates cancer genome evolution. Cancer Discov. 2014;4(2):175–85. 24436049 10.1158/2159-8290.CD-13-0285 PMC4293454

[CR15] Forbes SA, Bindal N, Bamford S, Cole C, Kok CY, Beare D, et al. COSMIC: mining complete cancer genomes in the catalogue of somatic mutations in cancer. Nucleic Acids Res. 2011;39:D945–50. 20952405 10.1093/nar/gkq929 PMC3013785

[CR16] Frankell AM, Dietzen M, Al Bakir M, Lim EL, Karasaki T, Ward S, et al. The evolution of lung cancer and impact of subclonal selection in TRACERx. Nature. 2023;616(7957):525–33. 37046096 10.1038/s41586-023-05783-5 PMC10115649

[CR17] Fujii Y, Sato Y, Suzuki H, Kakiuchi N, Yoshizato T, Lenis AT, et al. Molecular classification and diagnostics of upper urinary tract urothelial carcinoma. Cancer Cell. 2021;39(6):793-809.e8. 34129823 10.1016/j.ccell.2021.05.008 PMC9110171

[CR18] Gui Y, Guo G, Huang Y, Hu X, Tang A, Gao S, et al. Frequent mutations of chromatin remodeling genes in transitional cell carcinoma of the bladder. Nat Genet. 2011;43(9):875–8. 21822268 10.1038/ng.907 PMC5373841

[CR19] Hänzelmann S, Castelo R, Guinney J. GSVA: gene set variation analysis for microarray and RNA-Seq data. BMC Bioinform. 2013;14(1):7. 10.1186/1471-2105-14-7 PMC3618321 23323831

[CR20] Harrell FE. Regression modeling strategies: With applications to linear models, logistic and ordinal regression, and survival analysis. Cham: Springer; 2015 (Springer Series in Statistics). Available from: https://link.springer.com/10.1007/978-3-319-19425-7

[CR21] Hurst CD, Knowles MA. Mutational landscape of non-muscle-invasive bladder cancer. Urol Oncol: Seminars Orig Investig. 2022;40(7):295–303. 10.1016/j.urolonc.2018.10.015 30446444

[CR22] Hurst CD, Platt FM, Taylor CF, Knowles MA. Novel tumor subgroups of urothelial carcinoma of the bladder defined by integrated genomic analysis. Clin Cancer Res. 2012;18(21):5865–77. 22932667 10.1158/1078-0432.CCR-12-1807 PMC5081094

[CR23] Hurst CD, Alder O, Platt FM, Droop A, Stead LF, Burns JE, et al. Genomic subtypes of non-invasive bladder cancer with distinct metabolic profile and female gender bias in KDM6A mutation frequency. Cancer Cell. 2017;32(5):701-715.e7. 29136510 10.1016/j.ccell.2017.08.005 PMC5774674

[CR24] Jamal-Hanjani M, Wilson GA, McGranahan N, Birkbak NJ, Watkins TBK, Veeriah S, et al. Tracking the evolution of non–small-cell lung cancer. New Engl J Med. 2017;376(22):2109–21. 28445112 10.1056/NEJMoa1616288

[CR25] James AC, Gore JL. The costs of non-muscle invasive bladder cancer. Urol Clin N Am. 2013;40(2):261–9. 10.1016/j.ucl.2013.01.004 23540783

[CR26] Kamat AM, Li R, O’Donnell MA, Black PC, Roupret M, Catto JW, et al. Predicting response to intravesical Bacillus Calmette-Guérin immunotherapy: are we there yet? a systematic. Rev Eur Urol. 2018;73(5):738–48. 10.1016/j.eururo.2017.10.003 29055653

[CR27] Kang YC, Chen MH, Lin CY, Lin CY, Chen YT. Aristolochic acid-associated urinary tract cancers: an updated meta-analysis of risk and oncologic outcomes after surgery and systematic review of molecular alterations observed in human studies. Therapeutic Adv Drug Safety. 2021;1(12):2042098621997727. 10.1177/2042098621997727 PMC7989132 33815744

[CR28] Kent DG, Green AR. Order matters: the order of somatic mutations influences cancer evolution. Cold Spring Harb Perspect Med. 2017;7(4):a027060. 28096247 10.1101/cshperspect.a027060 PMC5378012

[CR29] Kim YJ, Ha YS, Kim SK, Yoon HY, Lym MS, Kim MJ, et al. Gene signatures for the prediction of response to Bacillus Calmette-Guérin immunotherapy in primary pT1 bladder cancers. Clin Cancer Res. 2010;16(7):2131–7. 20233890 10.1158/1078-0432.CCR-09-3323

[CR30] Kulkarni GS, Hakenberg OW, Gschwend JE, Thalmann G, Kassouf W, Kamat A, et al. An updated critical analysis of the treatment strategy for newly diagnosed high-grade T1 (previously T1G3) bladder cancer. Eur Urol. 2010;57(1):60–70. 19740595 10.1016/j.eururo.2009.08.024

[CR31] Kumar P, Henikoff S, Ng PC. Predicting the effects of coding non-synonymous variants on protein function using the SIFT algorithm. Nat Protoc. 2009;4(7):1073–81. 19561590 10.1038/nprot.2009.86

[CR32] Lawrence MS, Stojanov P, Polak P, Kryukov GV, Cibulskis K, Sivachenko A, et al. Mutational heterogeneity in cancer and the search for new cancer-associated genes. Nature. 2013;499(7457):214–8. 23770567 10.1038/nature12213 PMC3919509

[CR33] Li R, Du Y, Chen Z, Xu D, Lin T, Jin S, et al. Macroscopic somatic clonal expansion in morphologically normal human urothelium. Science. 2020;370(6512):82–9. 33004515 10.1126/science.aba7300

[CR34] Li J, Zhang Q, Tan Y, Duan Q, Sun T, Qi C. 120P The predictive value of LATS1 mutation for immune checkpoint inhibitors therapy in bladder cancer. Ann Oncol. 2022;1(33):S593.

[CR35] Lindskrog SV, Prip F, Lamy P, Taber A, Groeneveld CS, Birkenkamp-Demtröder K, et al. An integrated multi-omics analysis identifies prognostic molecular subtypes of non-muscle-invasive bladder cancer. Nat Commun. 2021;12(1):2301. 33863885 10.1038/s41467-021-22465-w PMC8052448

[CR36] Love MI, Huber W, Anders S. Moderated estimation of fold change and dispersion for RNA-seq data with DESeq2. Genome Biol. 2014;15:1–21. 10.1186/s13059-014-0550-8. 10.1186/s13059-014-0550-8 PMC4302049 25516281

[CR37] Martincorena I, Raine KM, Gerstung M, Dawson KJ, Haase K, Van Loo P, et al. Universal patterns of selection in cancer and somatic tissues. Cell. 2017;171(5):1029-1041.e21. 29056346 10.1016/j.cell.2017.09.042 PMC5720395

[CR38] McGranahan N, Favero F, De Bruin EC, Birkbak NJ, Szallasi Z, Swanton C. Clonal status of actionable driver events and the timing of mutational processes in cancer evolution. Sci Transl Med. 2015. 10.1126/scitranslmed.aaa1408. 25877892 10.1126/scitranslmed.aaa1408 PMC4636056

[CR39] McKenna A, Hanna M, Banks E, Sivachenko A, Cibulskis K, Kernytsky A, et al. The genome analysis toolkit: a MapReduce framework for analyzing next-generation DNA sequencing data. Genome Res. 2010;20(9):1297–303. 20644199 10.1101/gr.107524.110 PMC2928508

[CR40] Meghani K, Folgosa Cooley L, Piunti A, Meeks JJ. Role of chromatin modifying complexes and therapeutic opportunities in bladder cancer. Bladder Cancer. 2022;8(2):101–12. 35898580 10.3233/BLC-211609 PMC9278011

[CR41] Mermel CH, Schumacher SE, Hill B, Meyerson ML, Beroukhim R. Getz G. GISTIC2.0 facilitates sensitive and confident localization of the targets of focal somatic copy-number alteration in human cancers. Genome Biol. 2011. 10.1186/gb-2011-12-4-r41. 21527027 10.1186/gb-2011-12-4-r41 PMC3218867

[CR42] Miles J. Tolerance and variance inflation factor. In: Wiley StatsRef: statistics reference online. Hoboken: Wiley; 2014.

[CR43] Nordentoft I, Lamy P, Birkenkamp-Demtröder K, Shumansky K, Vang S, Hornshøj H, et al. Mutational context and diverse clonal development in early and late bladder cancer. Cell Rep. 2014;7(5):1649–63. 24835989 10.1016/j.celrep.2014.04.038

[CR44] Olislagers M, de Jong FC, Rutten VC, Boormans JL, Mahmoudi T, Zuiverloon TCM. Molecular biomarkers of progression in non-muscle-invasive bladder cancer—beyond conventional risk stratification. Nat Rev Urol. 2025;22(2):75–91. 39095581 10.1038/s41585-024-00914-7

[CR45] Patro R, Duggal G, Love MI, Irizarry RA, Kingsford C. Salmon provides fast and bias-aware quantification of transcript expression. Nat Methods. 2017;14(4):417–9. 28263959 10.1038/nmeth.4197 PMC5600148

[CR46] Pilala KM, Kotronopoulos G, Levis P, Giagkos GC, Stravodimos K, Vassilacopoulou D, et al. MIR145 core promoter methylation in pretreatment cell-free DNA: a liquid biopsy tool for muscle-invasive bladder cancer treatment outcome. JCO Precis Oncol. 2024;8:e2300414. 38579191 10.1200/PO.23.00414

[CR47] R Core Team. R: A language and environment for statistical computing [Internet]. Vienna, Austria: R Foundation for statistical computing; 2020. Available from: https://www.R-project.org/

[CR48] Robertson AG, Kim J, Al-Ahmadie H, Bellmunt J, Guo G, Cherniack AD, et al. Comprehensive molecular characterization of muscle-invasive bladder cancer. Cell. 2017;171(3):540-556.e25. 28988769 10.1016/j.cell.2017.09.007 PMC5687509

[CR49] Saadeldin MK, Shawer H, Mostafa A, Kassem NM, Amleh A, Siam R. New genetic variants of LATS1 detected in urinary bladder and colon cancer. Front Genetics. 2015. 10.3389/fgene.2014.00425. 10.3389/fgene.2014.00425 PMC4292772 25628642

[CR50] Sanchez-Vega F, Mina M, Armenia J, Chatila WK, Luna A, La KC, et al. Oncogenic signaling pathways in the cancer genome atlas. Cell. 2018;173(2):321-337.e10. 29625050 10.1016/j.cell.2018.03.035 PMC6070353

[CR51] Schwarz JM, Rödelsperger C, Schuelke M, Seelow D. MutationTaster evaluates disease-causing potential of sequence alterations. Nat Methods. 2010;7(8):575–6. 20676075 10.1038/nmeth0810-575

[CR52] Sjoberg DD, Vertosick E. D curves: decision curve analysis for model evaluation. 2024 [cited 2025 Feb 14]. Available from: https://cran.r-project.org/web/packages/dcurves/index.html

[CR53] Soneson C, Love MI, Robinson MD. Differential analyses for RNA-seq: transcript-level estimates improve gene-level inferences. F1000Research. 2016. Available from: https://f1000research.com/articles/4-1521 10.12688/f1000research.7563.1 PMC4712774 26925227

[CR54] Tarabichi M, Salcedo A, Deshwar AG, Ni Leathlobhair M, Wintersinger J, Wedge DC, et al. A practical guide to cancer subclonal reconstruction from DNA sequencing. Nat Methods. 2021;18(2):144–55. 33398189 10.1038/s41592-020-01013-2 PMC7867630

[CR55] Teh YW, Jordan MI, Beal MJ, Blei DM. Hierarchical dirichlet processes. J Am Stat Assoc. 2006;101(476):1566–81.

[CR56] Therneau TM, Foundation M. A package for survival analysis in S. 2015. p. 83.

[CR57] van der Vos KE, Vis DJ, Nevedomskaya E, Kim Y, Choi W, McConkey D, et al. Epigenetic profiling demarcates molecular subtypes of muscle-invasive bladder cancer. Sci Rep. 2020;10(1):10952. 32616859 10.1038/s41598-020-67850-5 PMC7331601

[CR58] Van Loo P, Nordgard SH, Lingjærde OC, Russnes HG, Rye IH, Sun W, Weigman VJ, Marynen P, Zetterberg A, Naume B, Perou CM. Allele-specific copy number analysis of tumors. Proc Natl Acad Sci. 2010;107(39):16910–5. 20837533 10.1073/pnas.1009843107 PMC2947907

[CR59] Vickers AJ, Elkin EB. Decision curve analysis: a novel method for evaluating prediction models. Med Decis Making. 2006;26(6):565–74. 17099194 10.1177/0272989X06295361 PMC2577036

[CR60] Weinstein JN, Akbani R, Broom BM, Wang W, Verhaak RGW, McConkey D, et al. Comprehensive molecular characterization of urothelial bladder carcinoma. Nature. 2014;507(7492):315–22. 24476821 10.1038/nature12965 PMC3962515

[CR61] Wong JKL, Aichmüller C, Schulze M, Hlevnjak M, Elgaafary S, Lichter P, et al. Association of mutation signature effectuating processes with mutation hotspots in driver genes and non-coding regions. Nat Commun. 2022;13(1):178. 35013316 10.1038/s41467-021-27792-6 PMC8748499

[CR62] Wu XR. Urothelial tumorigenesis: a tale of divergent pathways. Nat Rev Cancer. 2005;5(9):713–25. 16110317 10.1038/nrc1697

[CR63] Wu XR, Mendelsohn C, DeGraff DJ. Tumorigenicity of RTK/RAS in urothelium. Oncoscience. 2015;2(9):739–40. 26501074 10.18632/oncoscience.188 PMC4606002

[CR64] Xie N, Shen G, Gao W, Huang Z, Huang C, Fu L. Neoantigens: promising targets for cancer therapy. Sig Transduct Target Ther. 2023;8(1):1–38. 10.1038/s41392-022-01270-x PMC9816309 36604431

[CR65] Yang HY, Chen PC, Wang JD. Chinese herbs containing aristolochic acid associated with renal failure and urothelial carcinoma: a review from epidemiologic observations to causal inference. Biomed Res Int. 2014;27(2014):e569325. 10.1155/2014/569325 PMC4241283 25431765

[CR66] Yang HY, Yang CC, Wu CY, Wang LJ, Lu KL. Aristolochic acid and immunotherapy for urothelial carcinoma: directions for unmet needs. Int J Mol Sci. 2019;20(13):3162. 31261684 10.3390/ijms20133162 PMC6650931

[CR67] Yates LR, Campbell PJ. Evolution of the cancer genome. Nat Rev Genet. 2012;13(11):795–806. 23044827 10.1038/nrg3317 PMC3666082

[CR68] Zhang S, Lin T, Xiong X, Chen C, Tan P, Wei Q. Targeting histone modifiers in bladder cancer therapy—preclinical and clinical evidence. Nat Rev Urol. 2024;21(8):495–511. 38374198 10.1038/s41585-024-00857-z

[CR69] Zuiverloon TCM, Nieuweboer AJM, Vékony H, Kirkels WJ, Bangma CH, Zwarthoff EC. Markers predicting response to Bacillus Calmette-Guérin immunotherapy in high-risk bladder cancer patients: a systematic review. Eur Urol. 2012;61(1):128–45. 22000498 10.1016/j.eururo.2011.09.026

