# Supplementary material for: Genomic subtypes of non-muscle-invasive bladder cancer: guiding immunotherapy decision-making for patients exposed to aristolochic acid
Source: Mol Med. 2025 Apr 17;31:140. doi: 10.1186/s10020-025-01199-1 (PMC12004710; doi:10.1186/s10020-025-01199-1)
Supplement: Supplementary file 1 — Additional file 1 [file 10020_2025_1199_MOESM1_ESM.pdf]

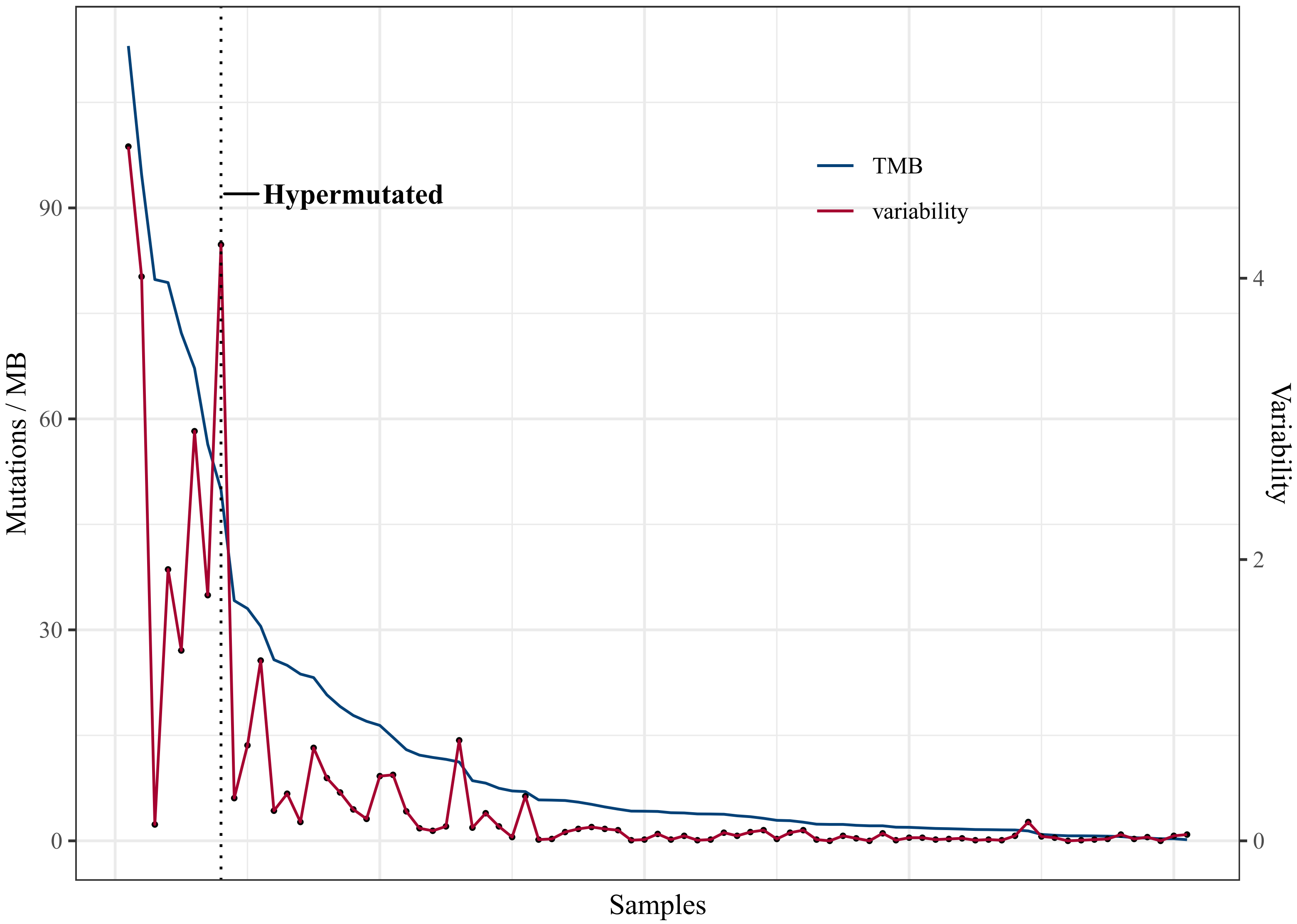

Figure S1: Mutation burden and their variability were depicted, the most drastic change point in the mutation rate was chosen for the cutoff to discriminate hypermutated cases from non-hypermutated ones.

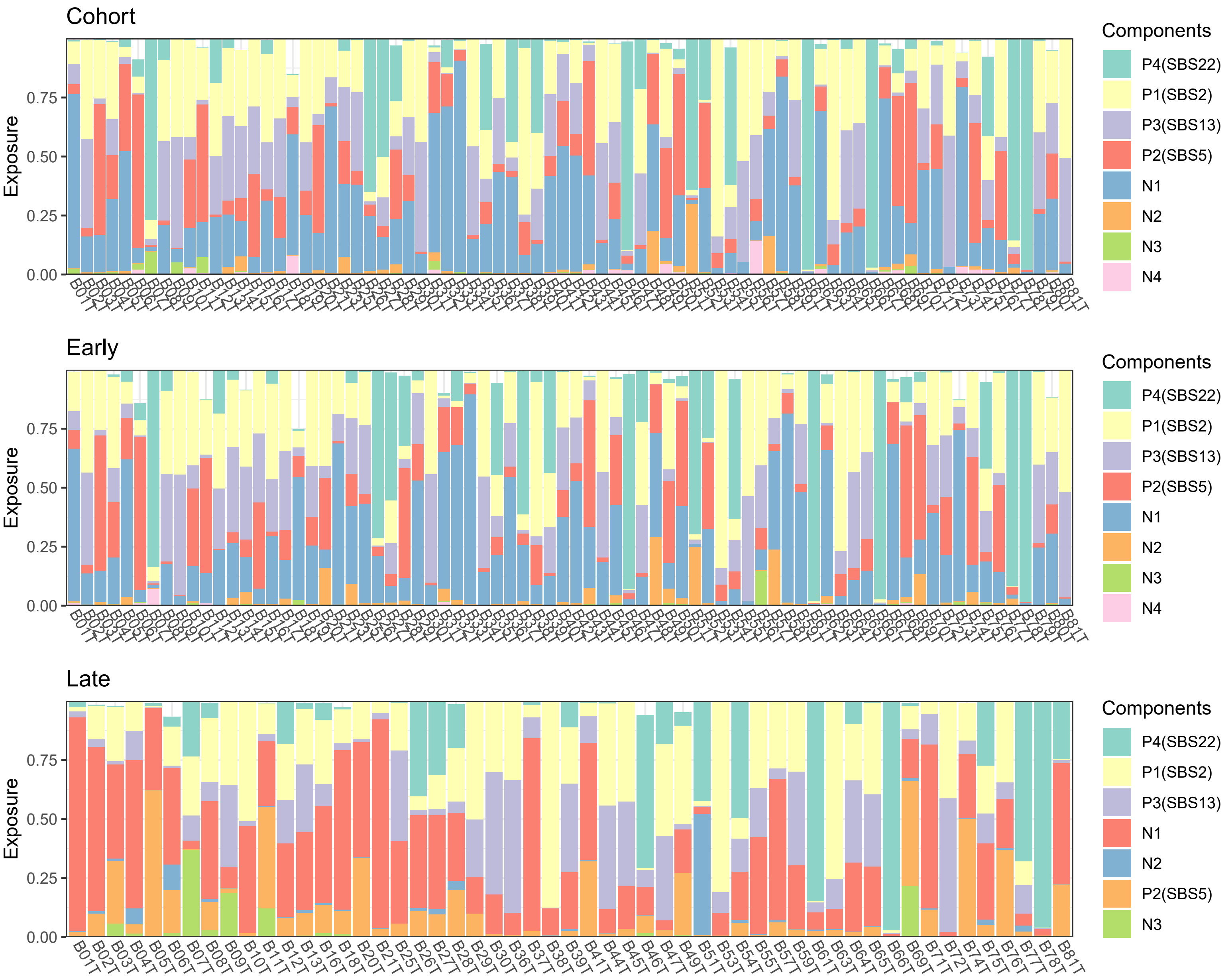

Figure S2: The exposures of signature extracted by Hierarchical Dirichlet Process (HDP) in reference to all mutations (Cohort), early mutations (Early) and late mutations (Late). The components labeled with "P" represent the priors.

Figure S3A

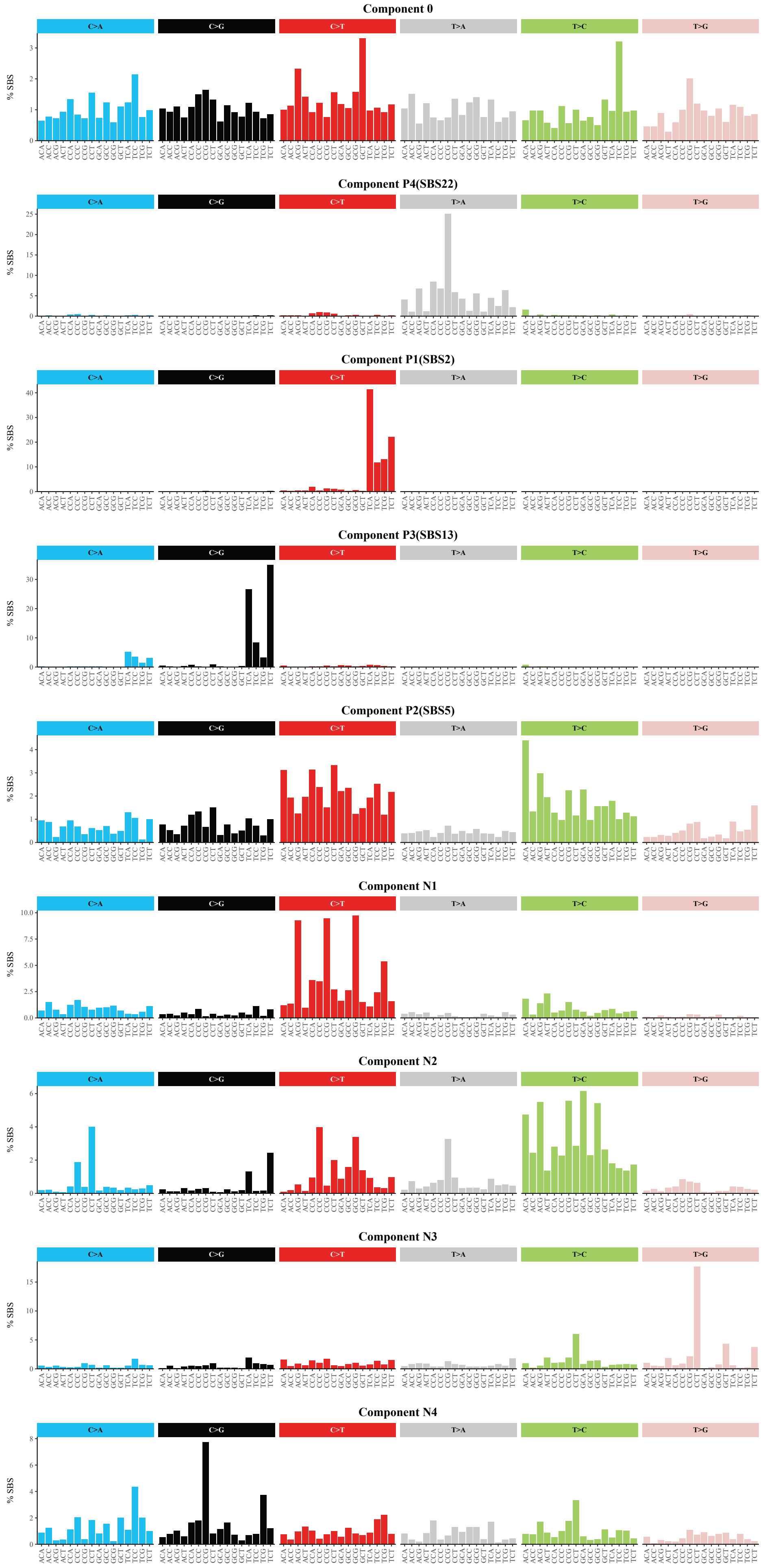

Component 0
Component P4(SBS22)
Component P1(SBS2)
Component P3(SBS13)
Component P2(SBS5)
Component N1
Component N2
Component N3
Component N4
C>A
C>G
C>T
T>A
T>C
T>G
% SBS

Figure S3B

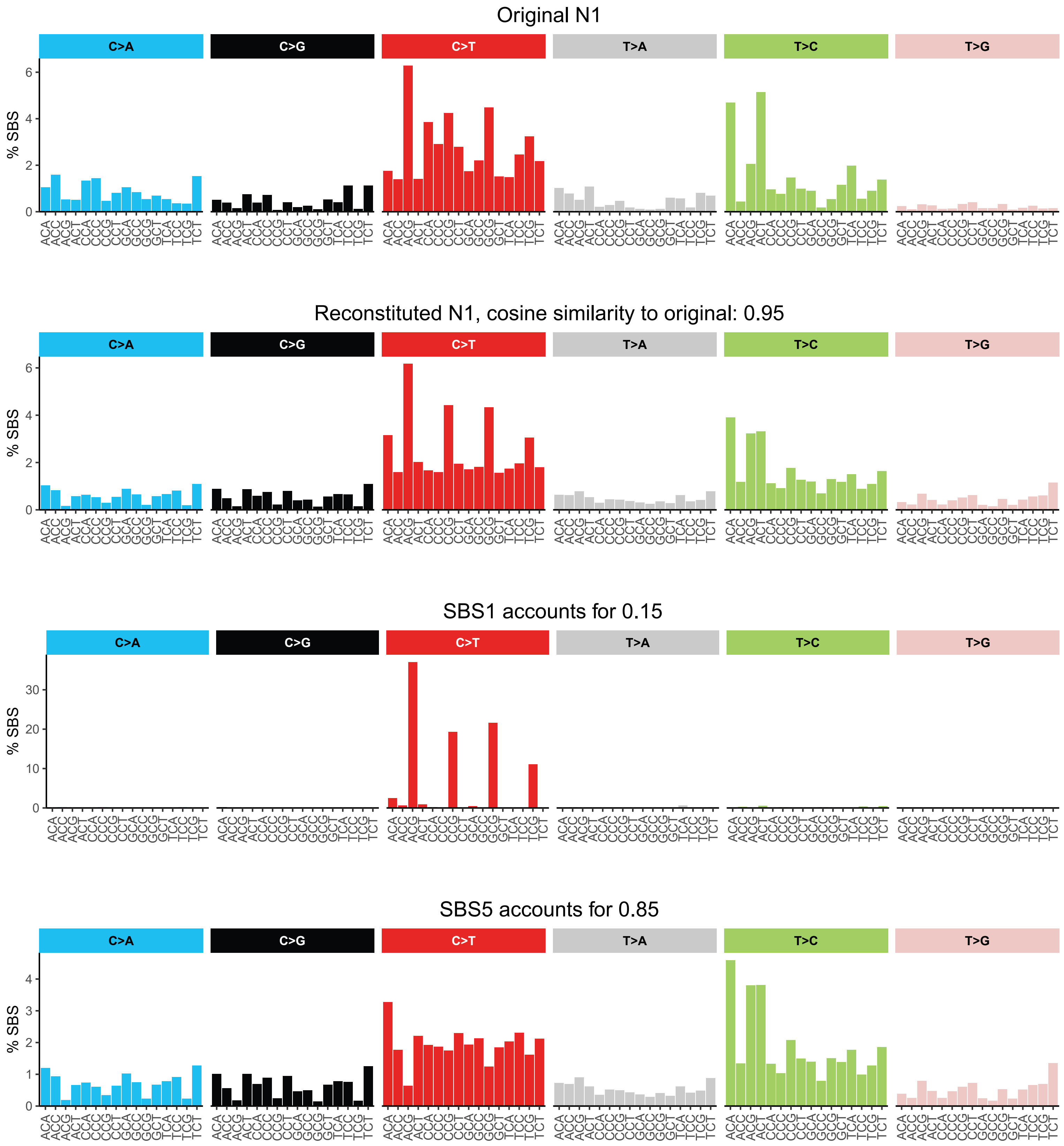

Original N1
Reconstituted N1, cosine similarity to original: 0.95
SBS1 accounts for 0.15
SBS5 accounts for 0.85
C>A
C>G
C>T
T>A
T>C
T>G
% SBS
ACA
ACC
ACG
ACT
CCA
CCC
CCG
CCT
GCA
GCC
GCG
GCT
TCA
TCC
TCG
TCT

Figure S3C

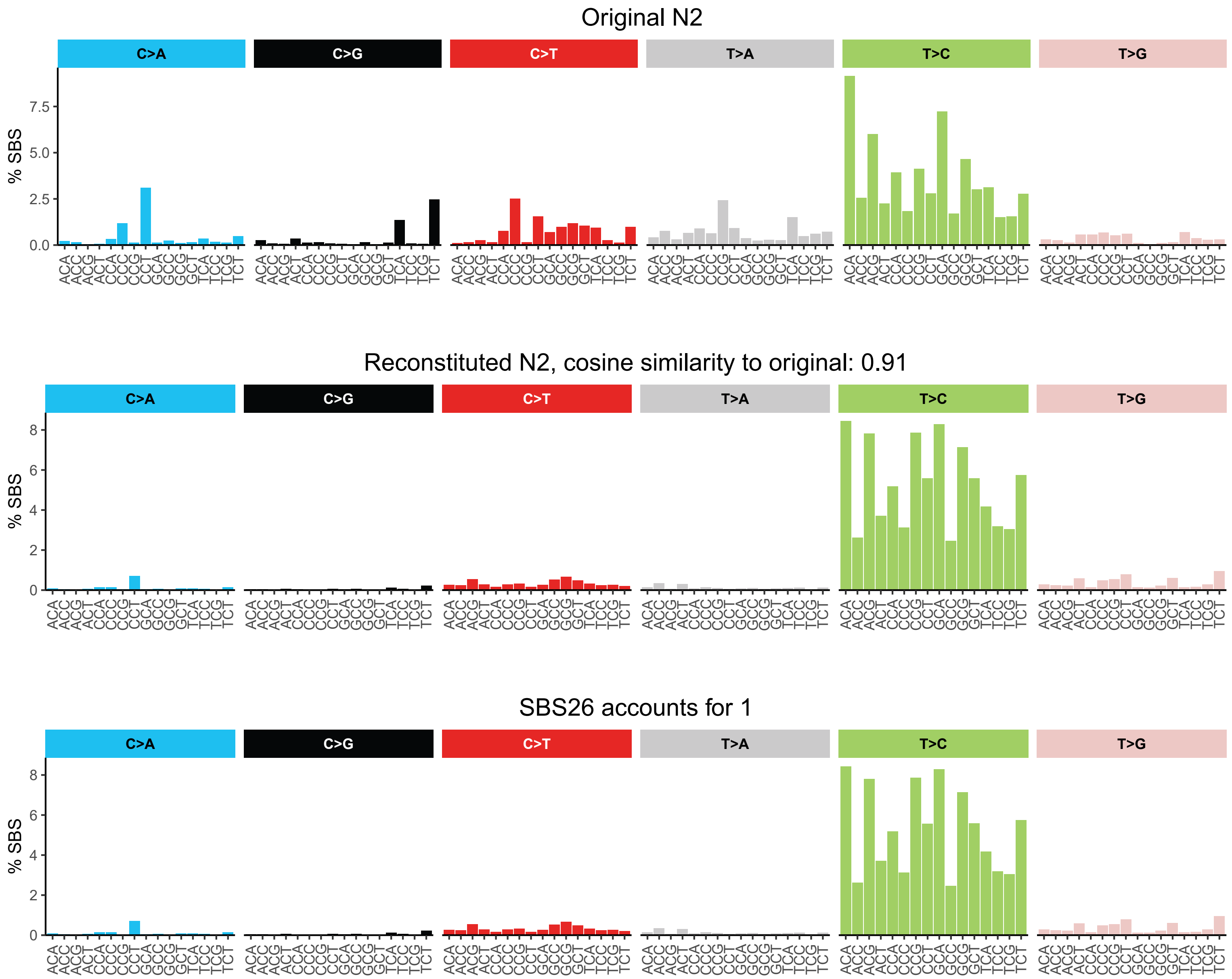
Original N2
Reconstituted N2, cosine similarity to original: 0.91
SBS26 accounts for 1
C>A
C>G
C>T
T>A
T>C
T>G
% SBS
ACA
ACC
ACG
ACT
CCA
CCC
CCG
CCT
GCA
GCC
GCG
GCT
TCA
TCC
TCG
TCT

Figure S3D

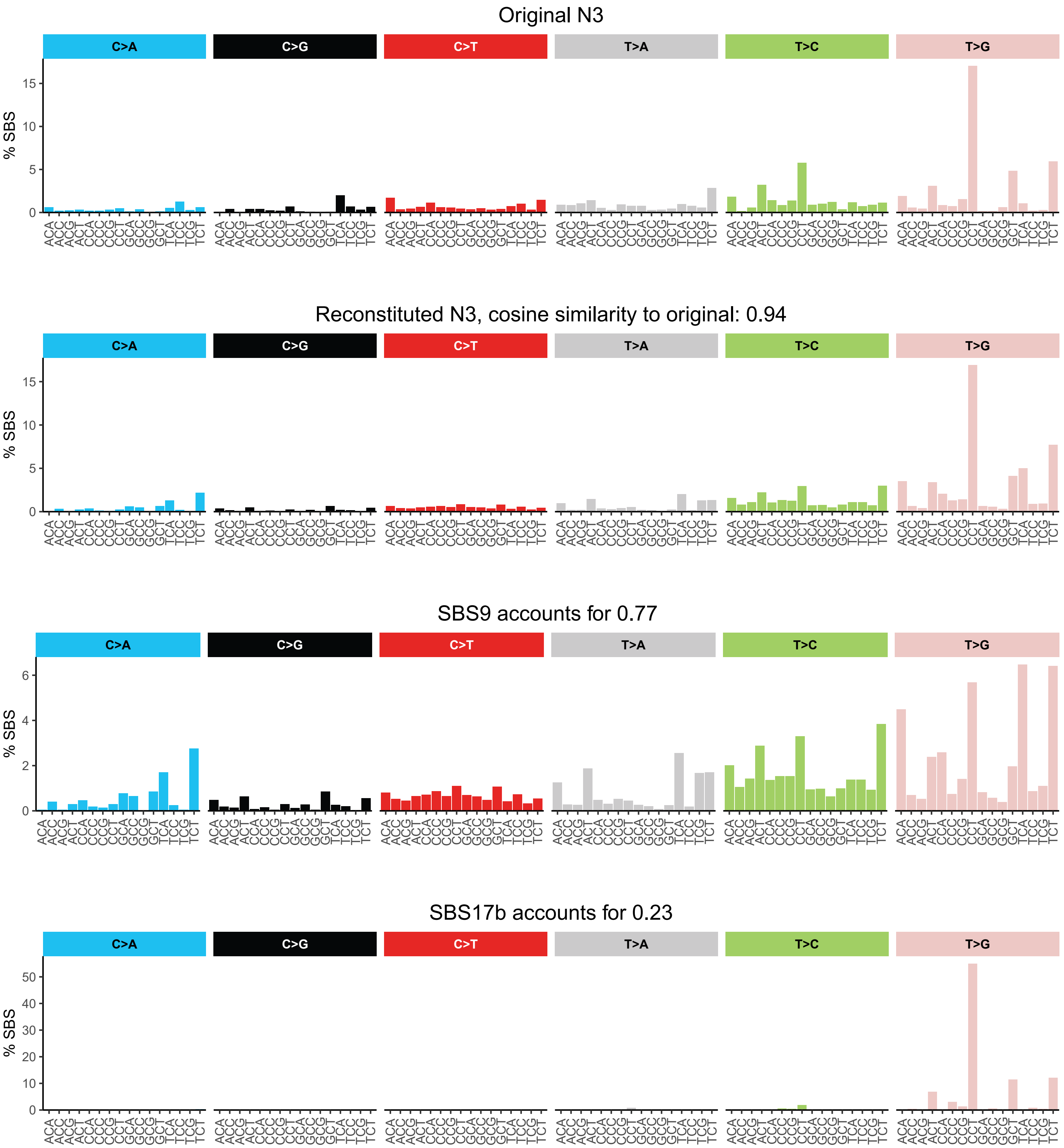
Original N3
Reconstituted N3, cosine similarity to original: 0.94
SBS9 accounts for 0.77
SBS17b accounts for 0.23
C>A
C>G
C>T
T>A
T>C
T>G
% SBS
ACA
ACC
ACG
ACT
CCA
CCC
CCG
CCT
GCA
GCC
GCG
GCT
TCA
TCC
TCG
TCT

Figure S3E

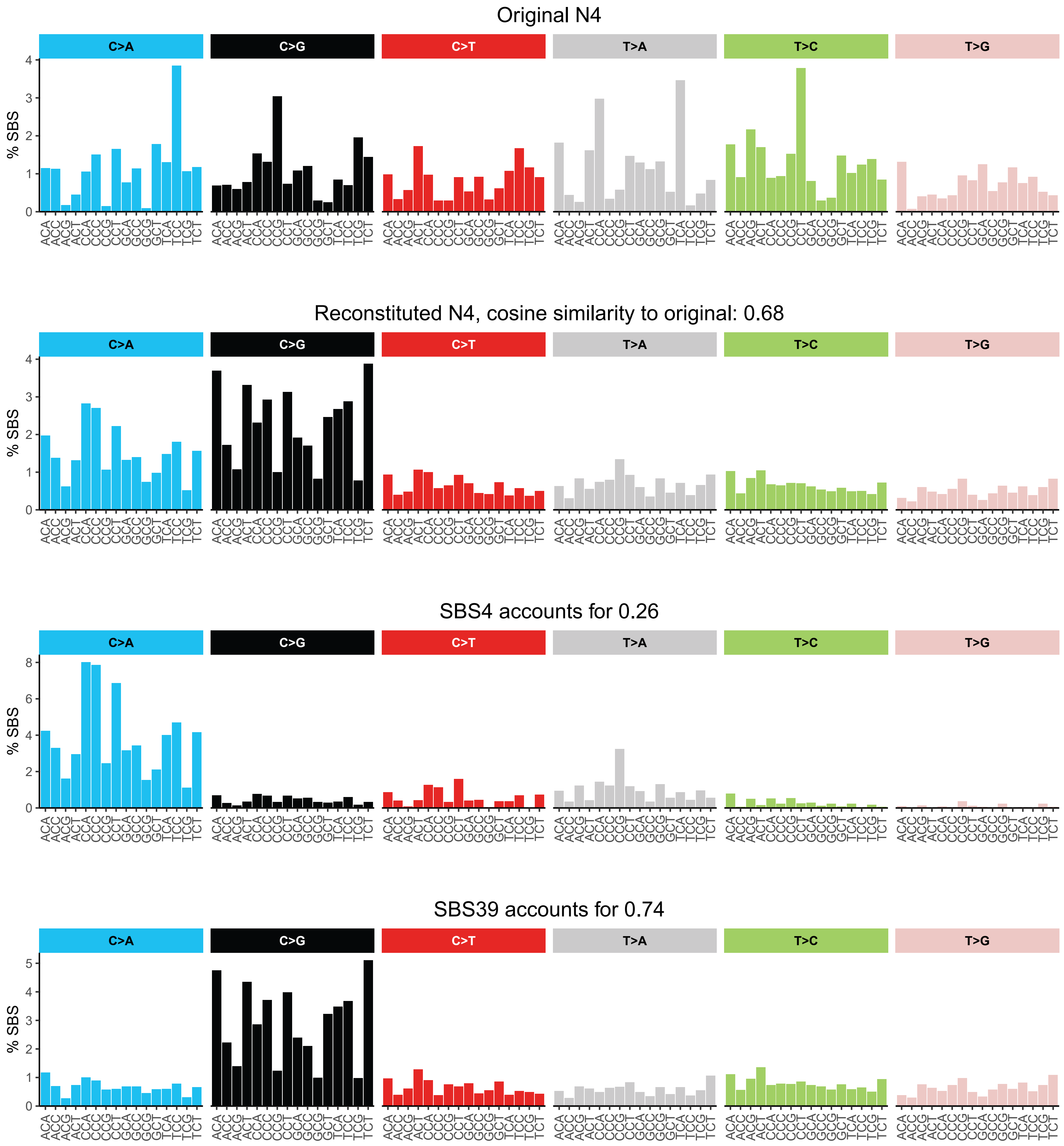

Figure S3: The mutational signatures were extracted de novo based on all SNVs using the HDP and expectation maximization (EM) algorithm. (A) The mutational spectrum of SBS signature, extracted using the HDP was depicted. (B-E) Unknown signatures were explained by known signatures in the COSMIC database using the EM algorithm. If the reconstructed signature showed a cosine similarity greater than 0.9 with the observed signature, it was recognized as a combination of the identified pair. In such cases, the exposure of the observed signature was split based on the weights provided by the EM algorithm for further analyses.

Figure S4A

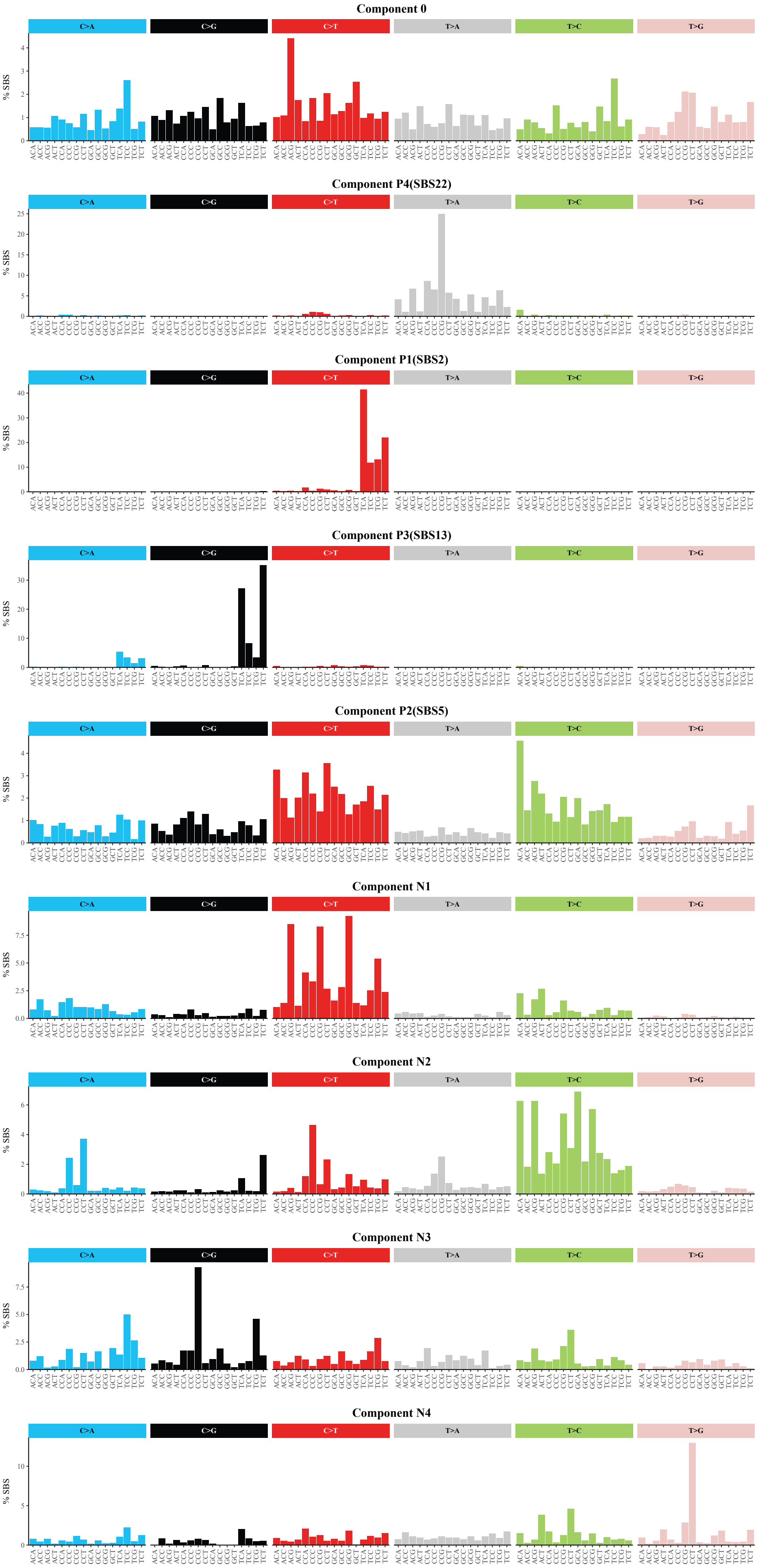
Component 0
Component P4(SBS22)
Component P1(SBS2)
Component P3(SBS13)
Component P2(SBS5)
Component N1
Component N2
Component N3
Component N4
C>A
C>G
C>T
T>A
T>C
T>G
% SBS

Figure S4B

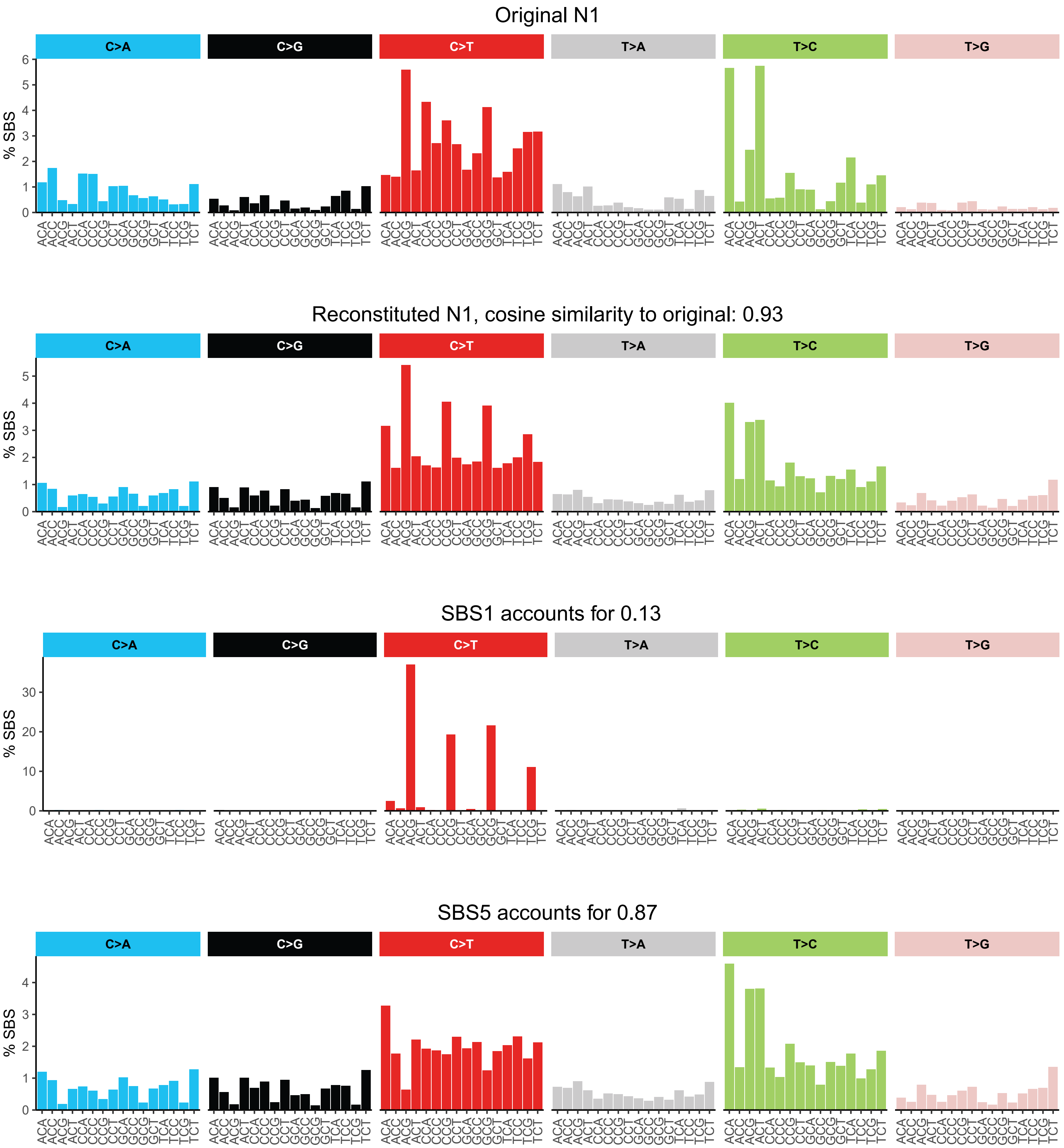

Original N1
C>A
C>G
C>T
T>A
T>C
T>G
% SBS
0
1
2
3
4
5
6
ACA
ACC
ACG
ACT
CCA
CCC
CCG
CCT
GCA
GCC
GCG
GCT
TCA
TCC
TCG
TCT
Reconstituted N1, cosine similarity to original: 0.93
SBS1 accounts for 0.13
10
20
30
SBS5 accounts for 0.87

Figure S4C

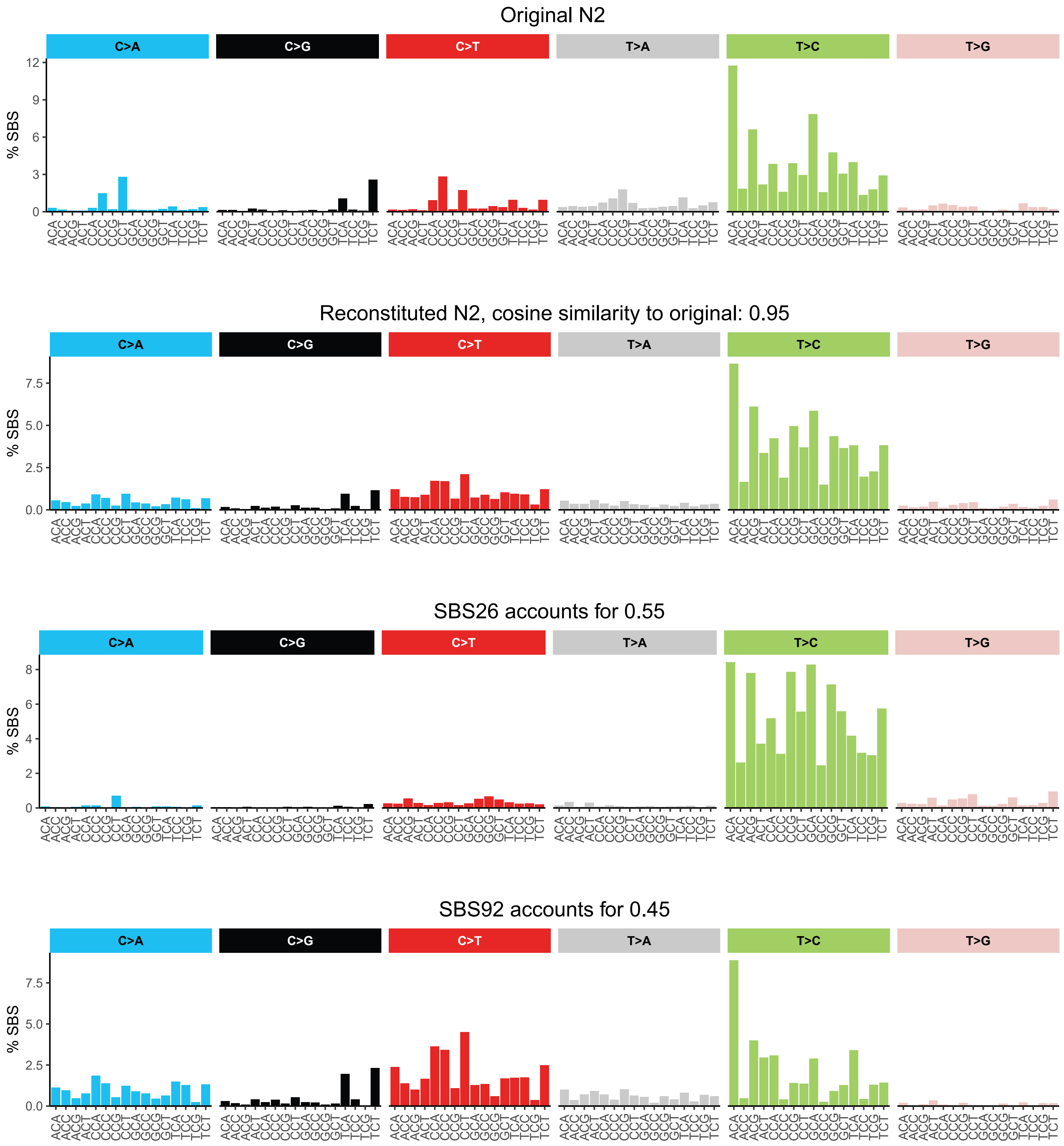

Original N2
C>A
C>G
C>T
T>A
T>C
T>G
% SBS
Reconstituted N2, cosine similarity to original: 0.95
SBS26 accounts for 0.55
SBS92 accounts for 0.45
ACA
ACC
ACG
ACT
CCA
CCC
CCG
CCT
GCA
GCC
GCG
GCT
TCA
TCC
TCG
TCT

Figure S4D

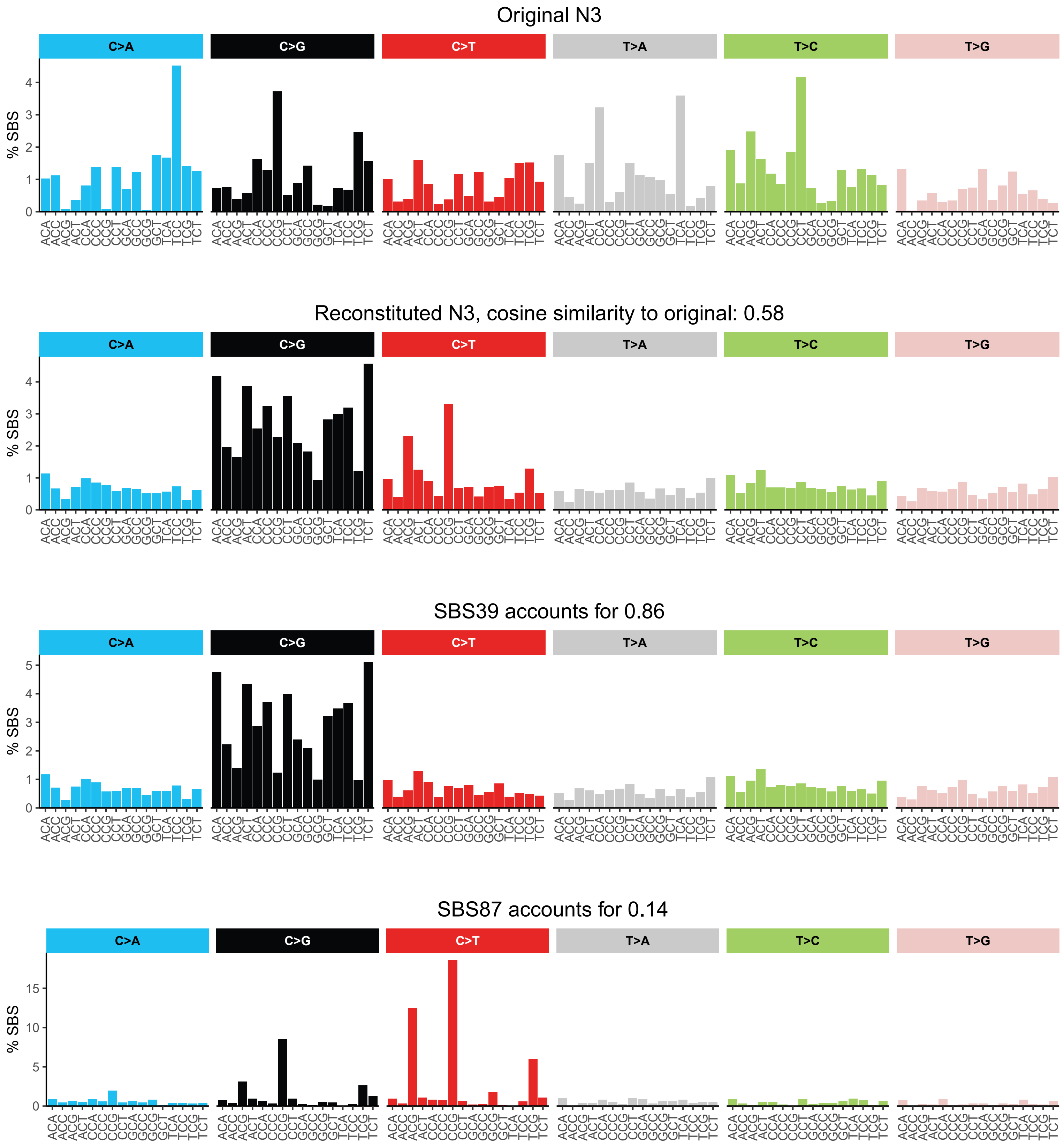
Original N3
Reconstituted N3, cosine similarity to original: 0.58
SBS39 accounts for 0.86
SBS87 accounts for 0.14
C>A
C>G
C>T
T>A
T>C
T>G
% SBS
ACA ACC ACG ACT CCA CCC CCG CCT GCA GCC GCG GCT TCA TCC TCG TCT

Figure S4E

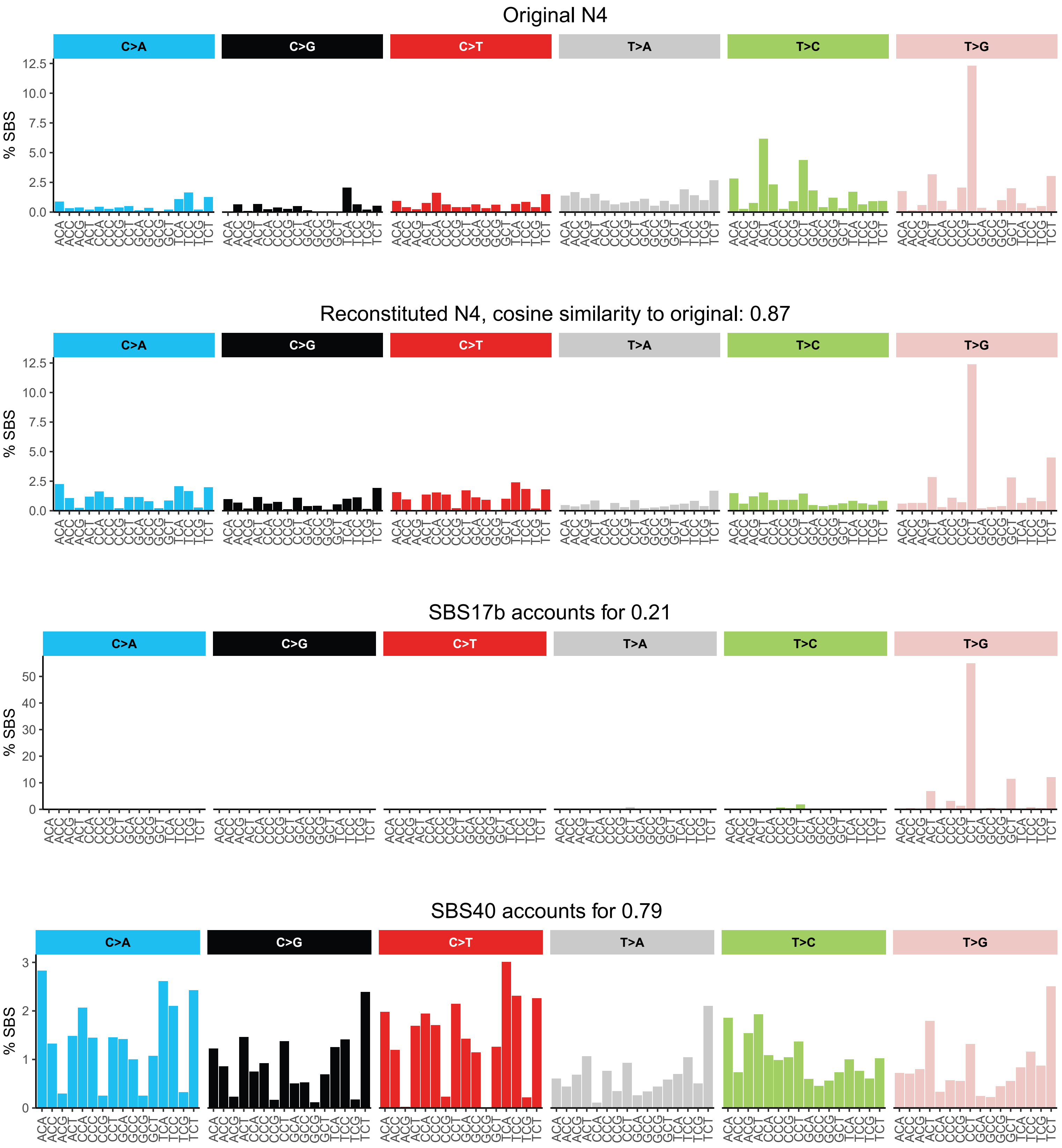

Figure S4: The mutational signatures were extracted de novo based on early SNVs using the HDP and expectation maximization (EM) algorithm. (A) The mutational spectrum of SBS signature, extracted using the HDP was depicted. (B-E)Unknown signatures were explained by known signatures in the COSMIC database using the EM algorithm. If the reconstructed signature showed a cosine similarity greater than 0.9 with the observed signature, it was recognized as a combination of the identified pair. In such cases, the exposure of the observed signature was split based on the weights provided by the EM algorithm for further analyses.

Figure S5A

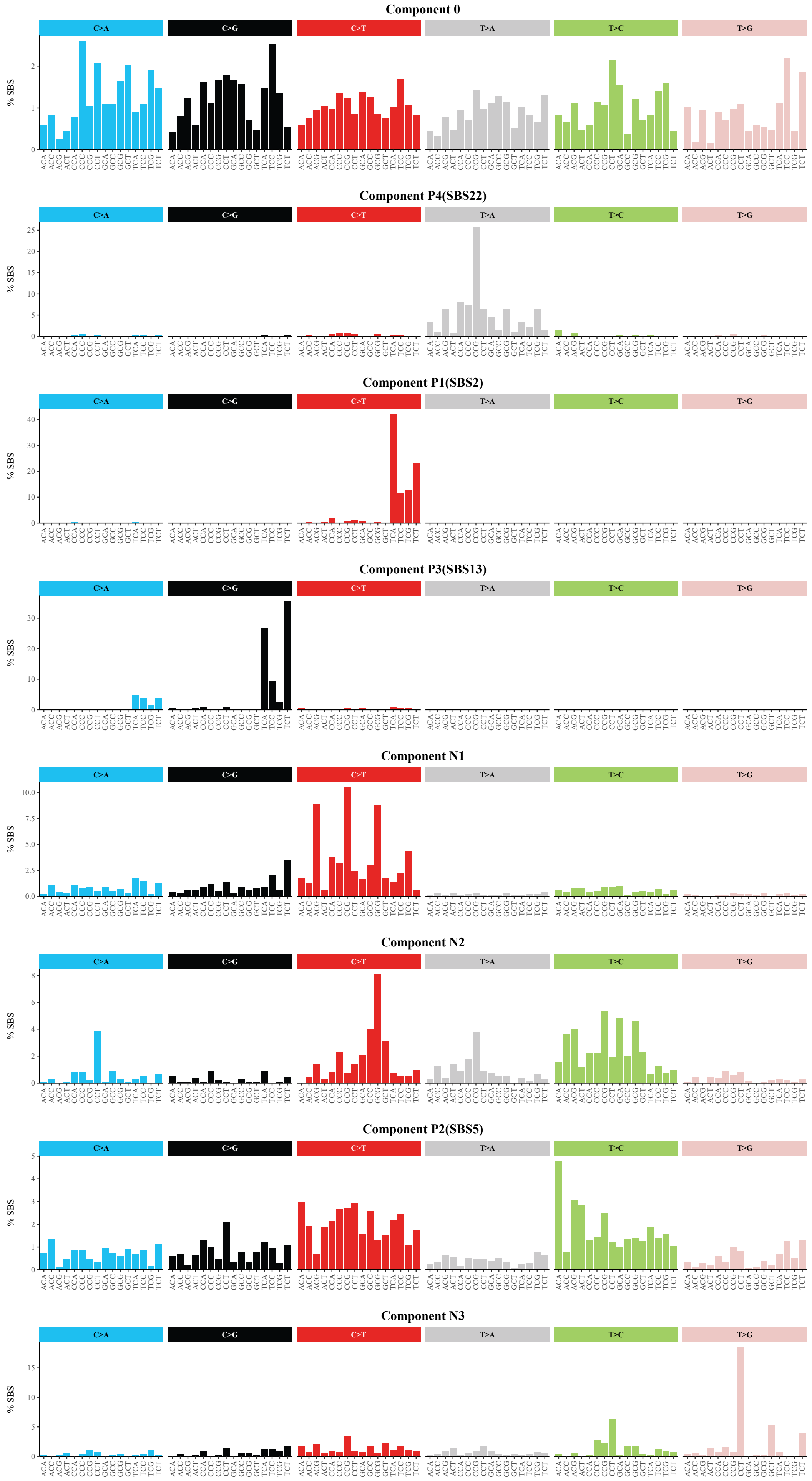
Component 0
Component P4(SBS22)
Component P1(SBS2)
Component P3(SBS13)
Component N1
Component N2
Component P2(SBS5)
Component N3
C>A
C>G
C>T
T>A
T>C
T>G
% SBS
ACA ACC ACG ACT CCA CCC CCG CCT GCA GCC GCG GCT TCA TCC TCG TCT

Figure S5B

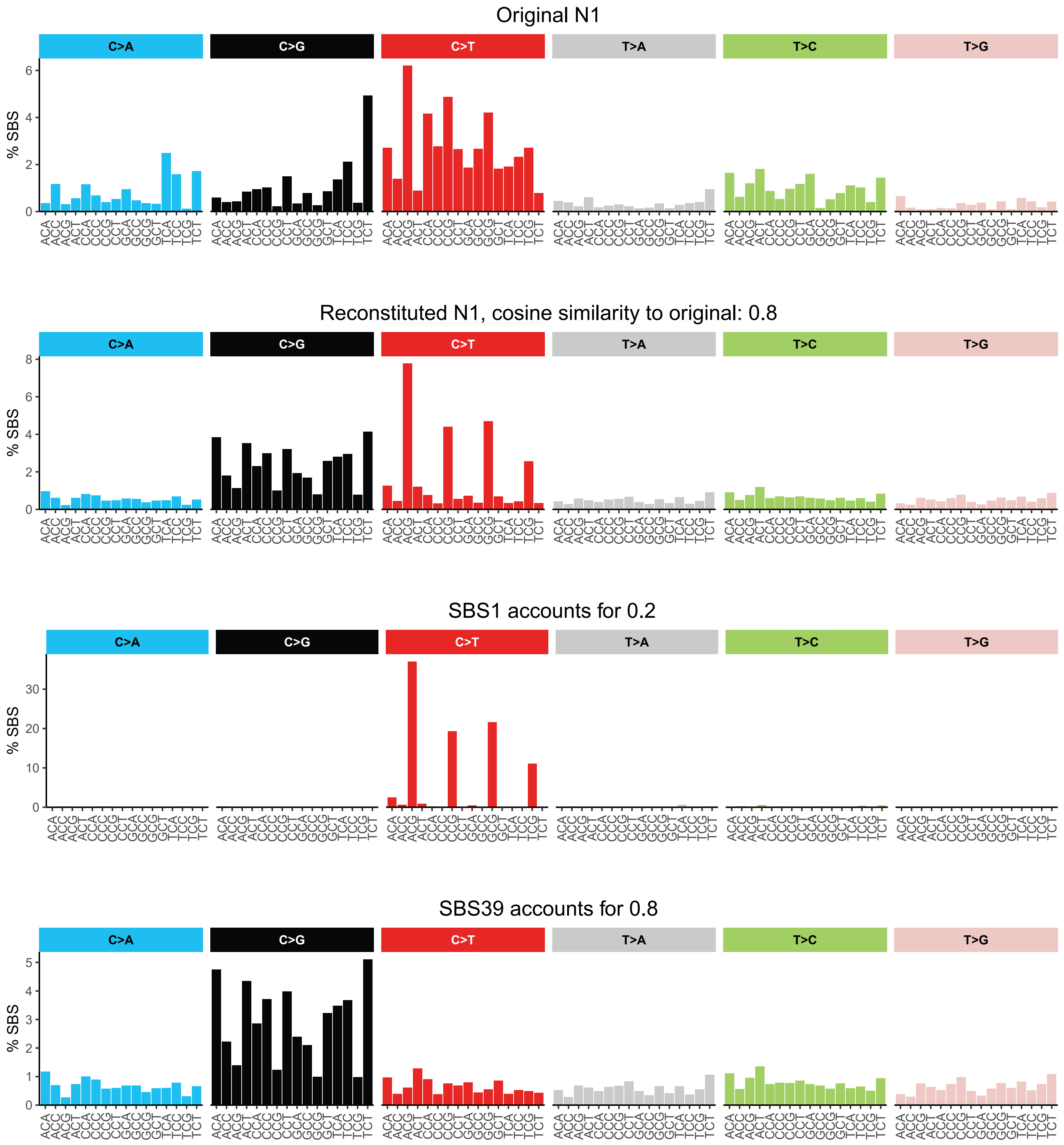
Original N1
C>A
C>G
C>T
T>A
T>C
T>G
% SBS
Reconstituted N1, cosine similarity to original: 0.8
SBS1 accounts for 0.2
SBS39 accounts for 0.8
ACA
ACC
ACG
ACT
CCA
CCC
CCG
CCT
GCA
GCC
GCG
GCT
TCA
TCC
TCG
TCT

Figure S5C

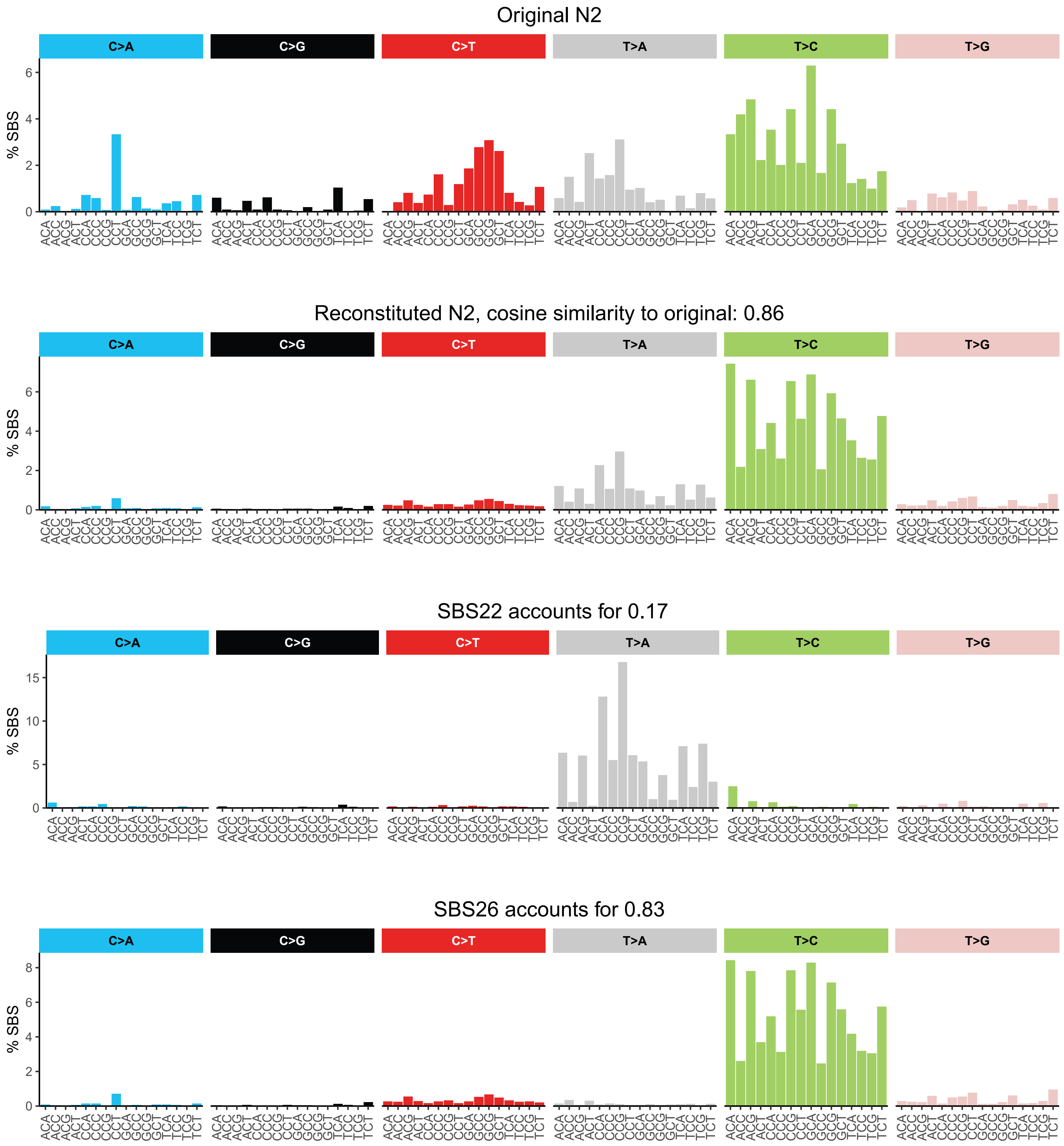

Original N2
C>A
C>G
C>T
T>A
T>C
T>G
% SBS
Reconstituted N2, cosine similarity to original: 0.86
SBS22 accounts for 0.17
SBS26 accounts for 0.83
ACA
ACC
ACG
ACT
CCA
CCC
CCG
CCT
GCA
GCC
GCG
GCT
TCA
TCC
TCG
TCT

Figure S5D

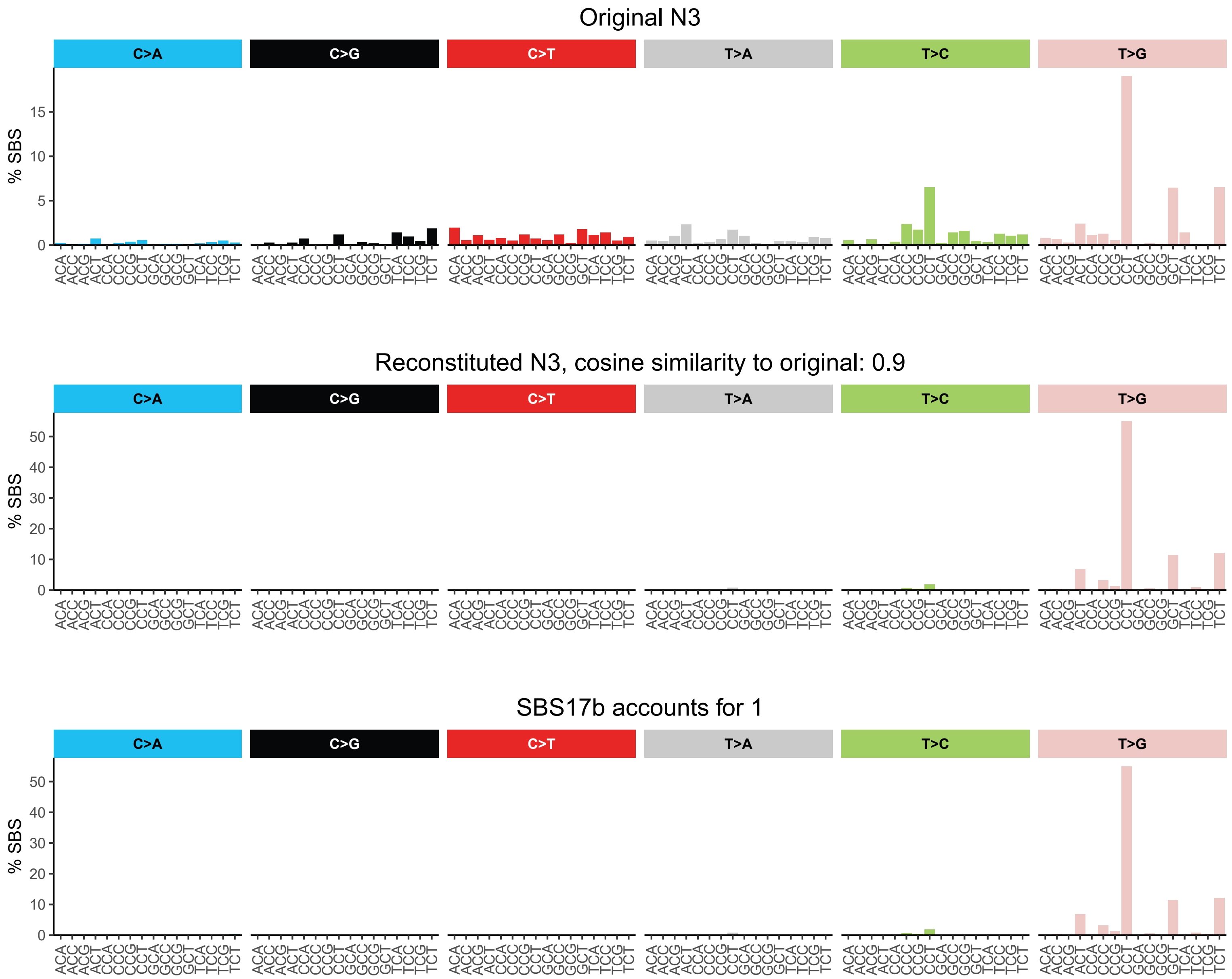

Figure S5: The mutational signatures were extracted de novo based on late SNVs using the HDP and expectation maximization (EM) algorithm. (A) The mutational spectrum of SBS signature, extracted using the HDP was depicted. (B-E) Unknown signatures were explained by known signatures in the COSMIC database using the EM algorithm. If the reconstructed signature showed a cosine similarity greater than 0.9 with the observed signature, it was recognized as a combination of the identified pair. In such cases, the exposure of the observed signature was split based on the weights provided by the EM algorithm for further analyses.

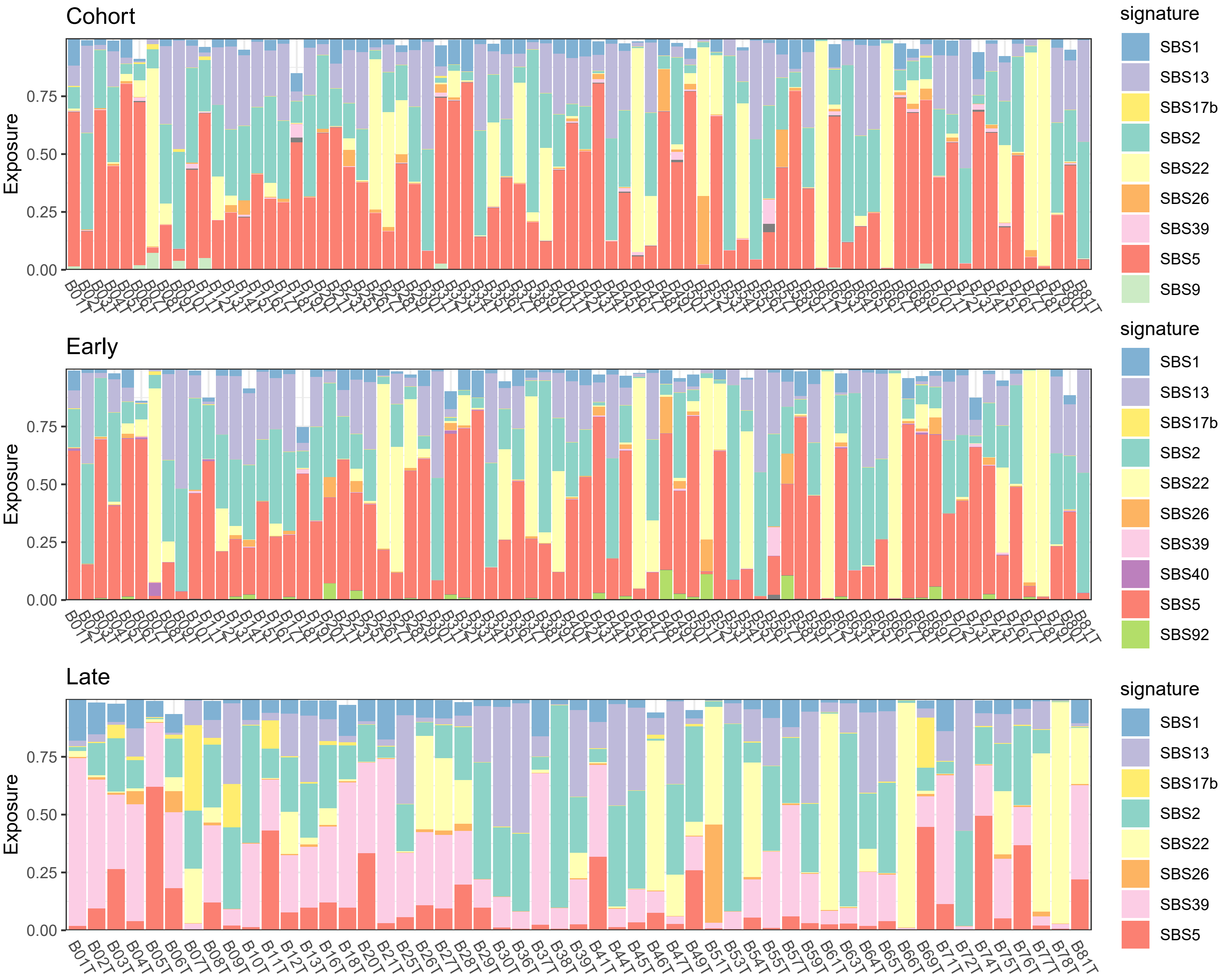

Figure S6: The exposures of the signatures, which were split by the EM algorithm, are presented for all mutations (Cohort), early mutations (Early), and late mutations (Late).

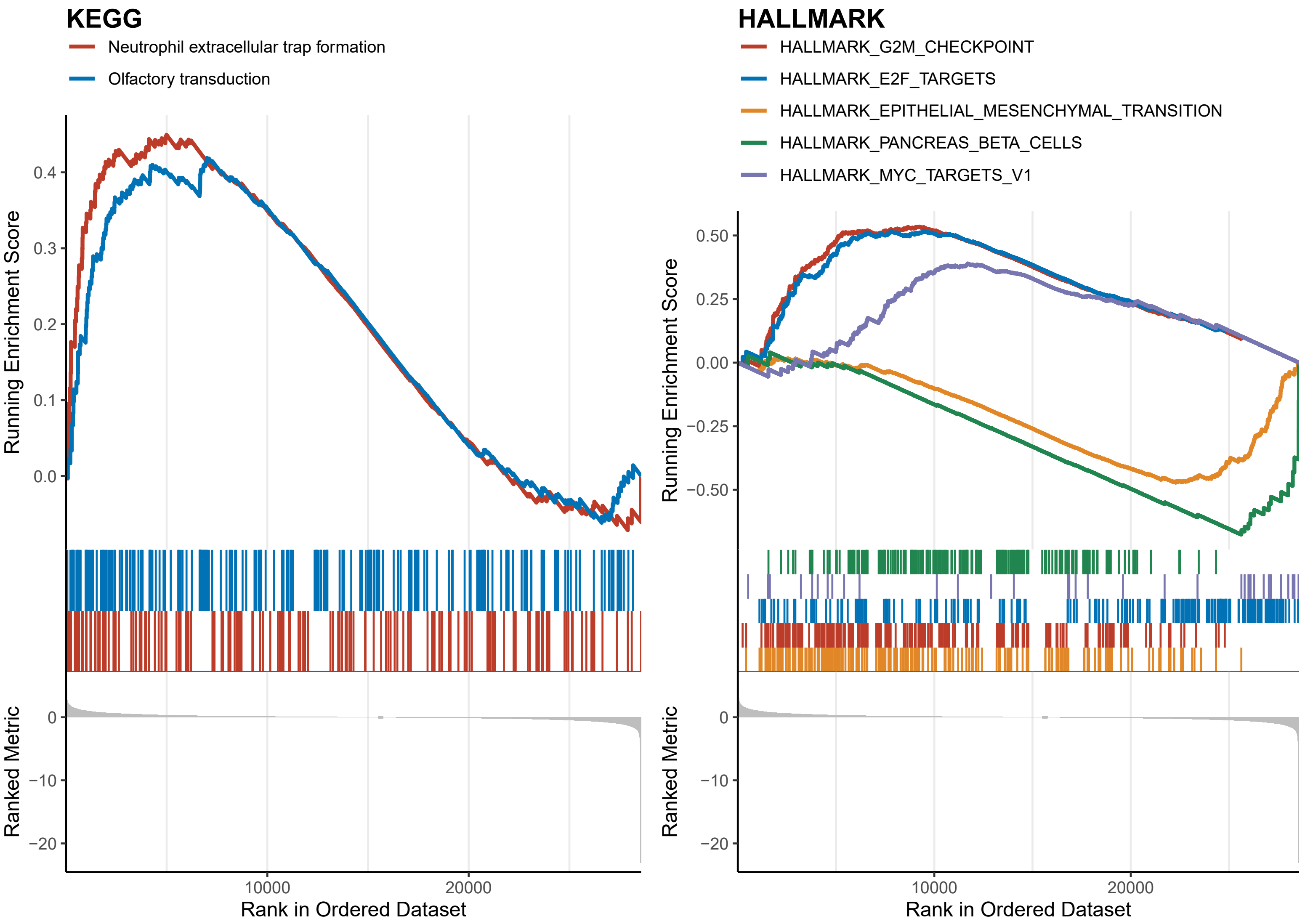

Figure S7: Exploration of the function of SBS22 in NMIBC.

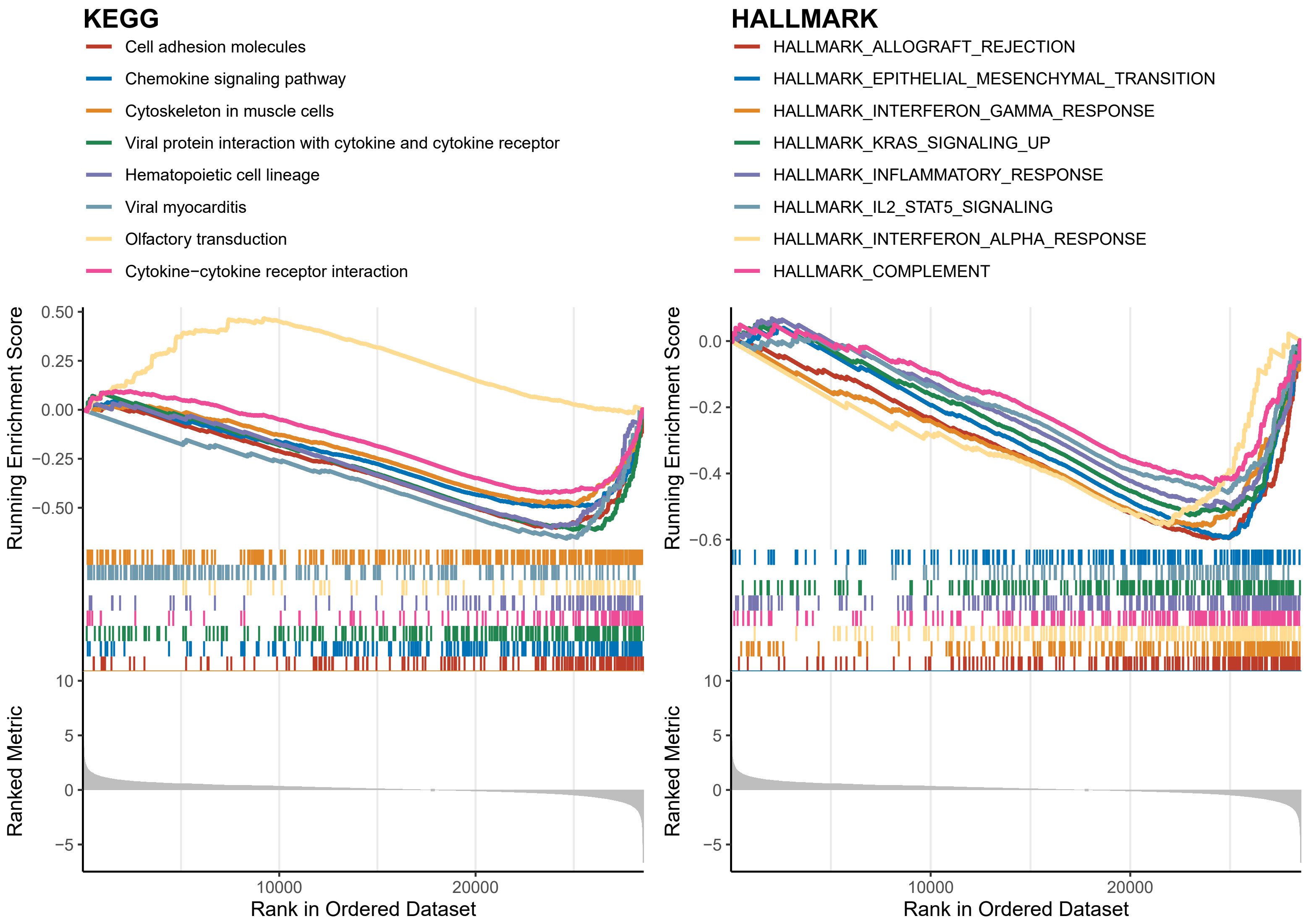

Figure S8: Exploration of the function of late SBS39 in NMIBC.

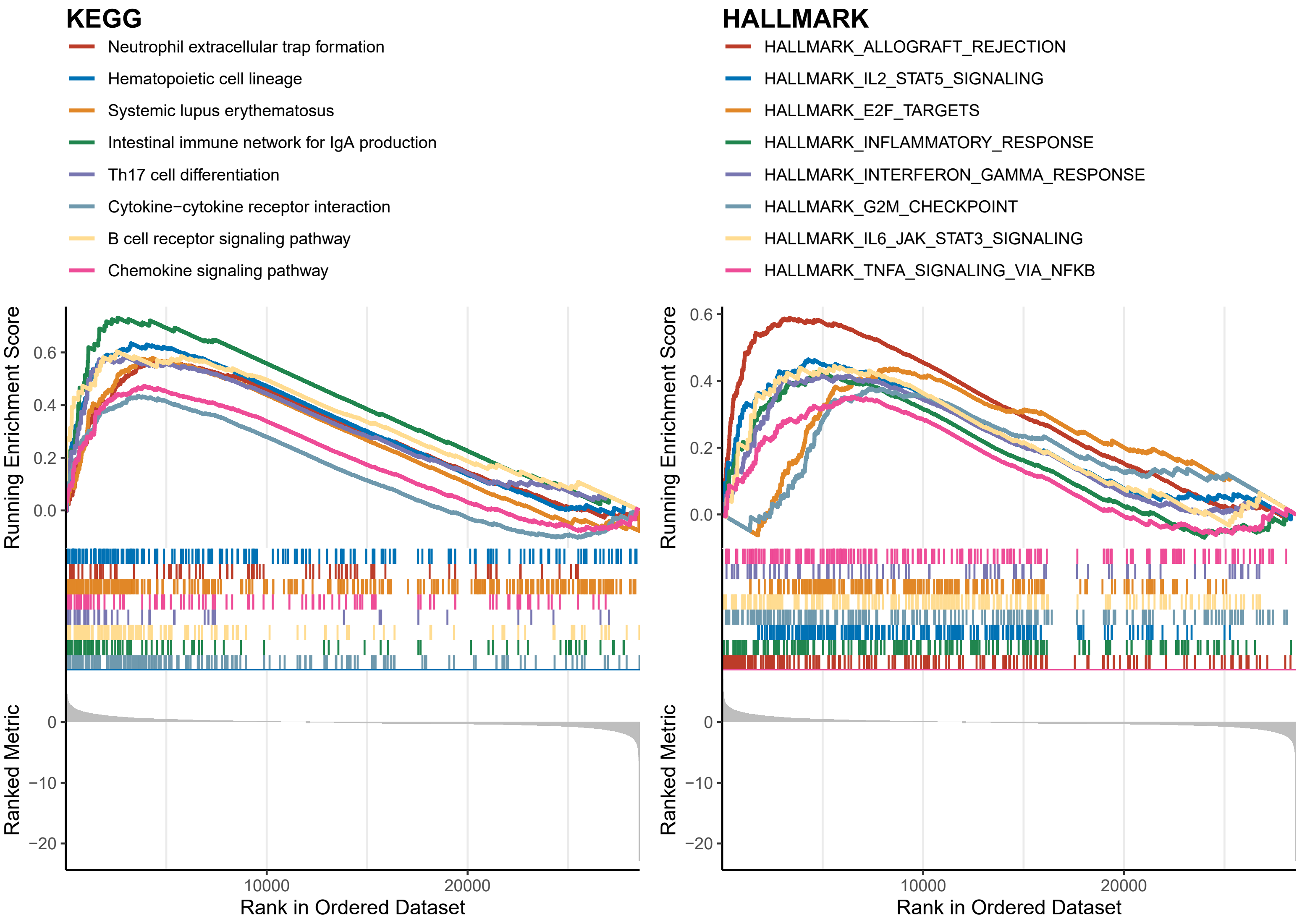

Figure S9: Exploration of the function of LATS1 early mutations in NMIBC.

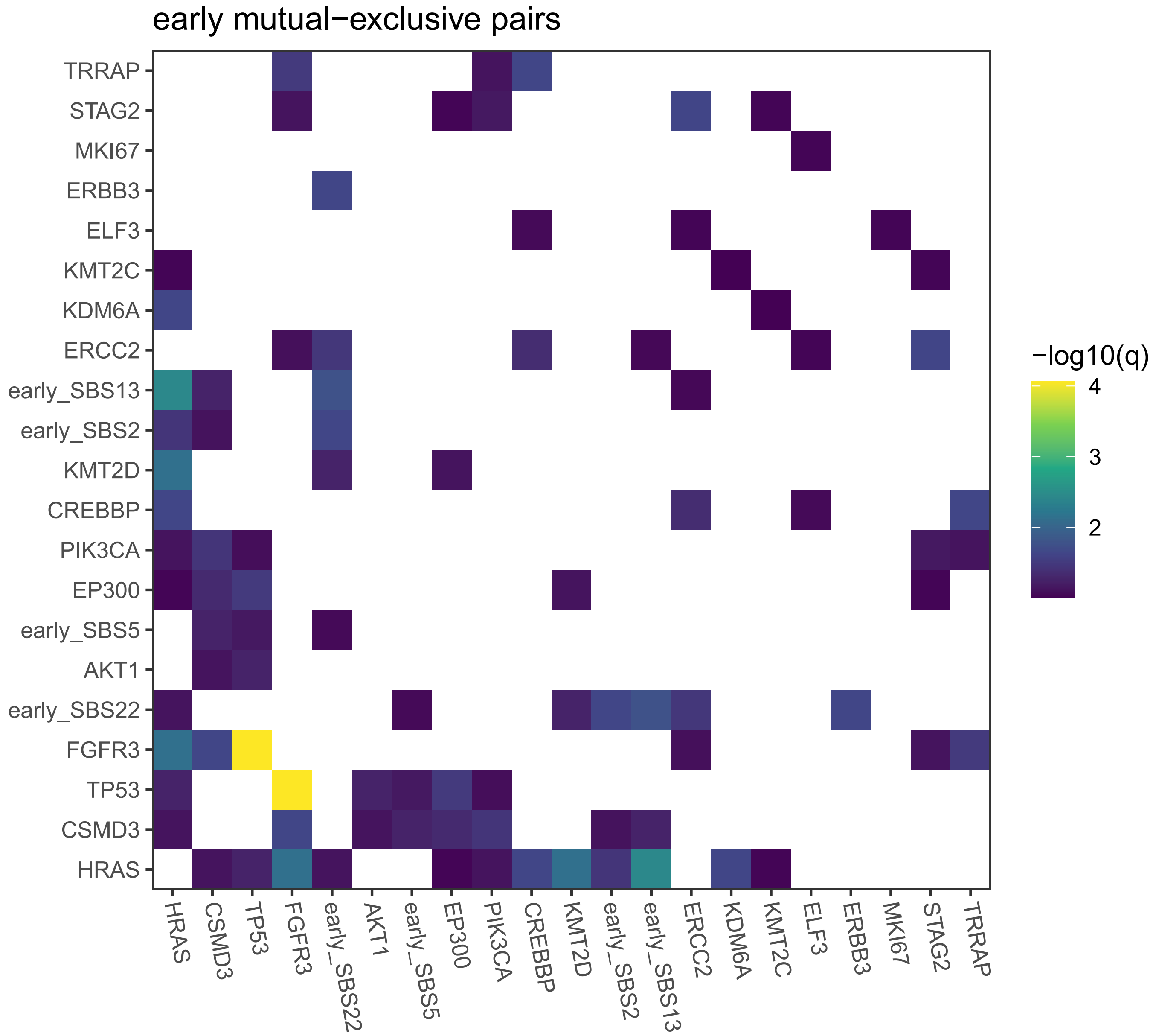

Figure S10: Mutual exclusivity relationships among early genomic events defined by DISCOVER. Only significant mutual exclusivity pairs are shown.

Figure S11A

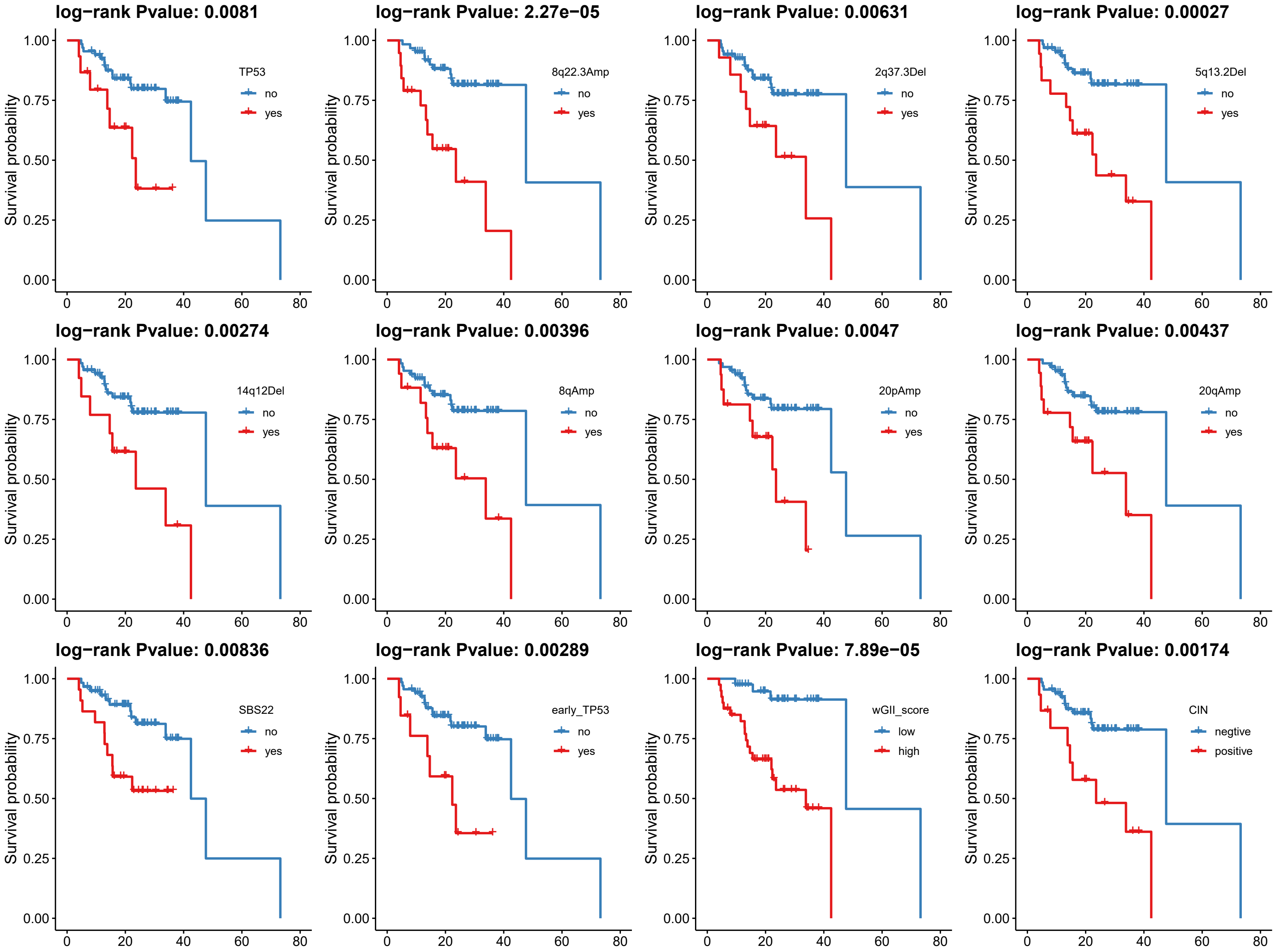
log−rank Pvalue: 0.0081
TP53
no
yes
log−rank Pvalue: 2.27e−05
8q22.3Amp
no
yes
log−rank Pvalue: 0.00631
2q37.3Del
no
yes
log−rank Pvalue: 0.00027
5q13.2Del
no
yes
log−rank Pvalue: 0.00274
14q12Del
no
yes
log−rank Pvalue: 0.00396
8qAmp
no
yes
log−rank Pvalue: 0.0047
20pAmp
no
yes
log−rank Pvalue: 0.00437
20qAmp
no
yes
log−rank Pvalue: 0.00836
SBS22
no
yes
log−rank Pvalue: 0.00289
early_TP53
no
yes
log−rank Pvalue: 7.89e−05
wGII_score
low
high
log−rank Pvalue: 0.00174
CIN
negtive
positive
Survival probability
1.00
0.75
0.50
0.25
0.00
0
20
40
60
80

Figure S11B

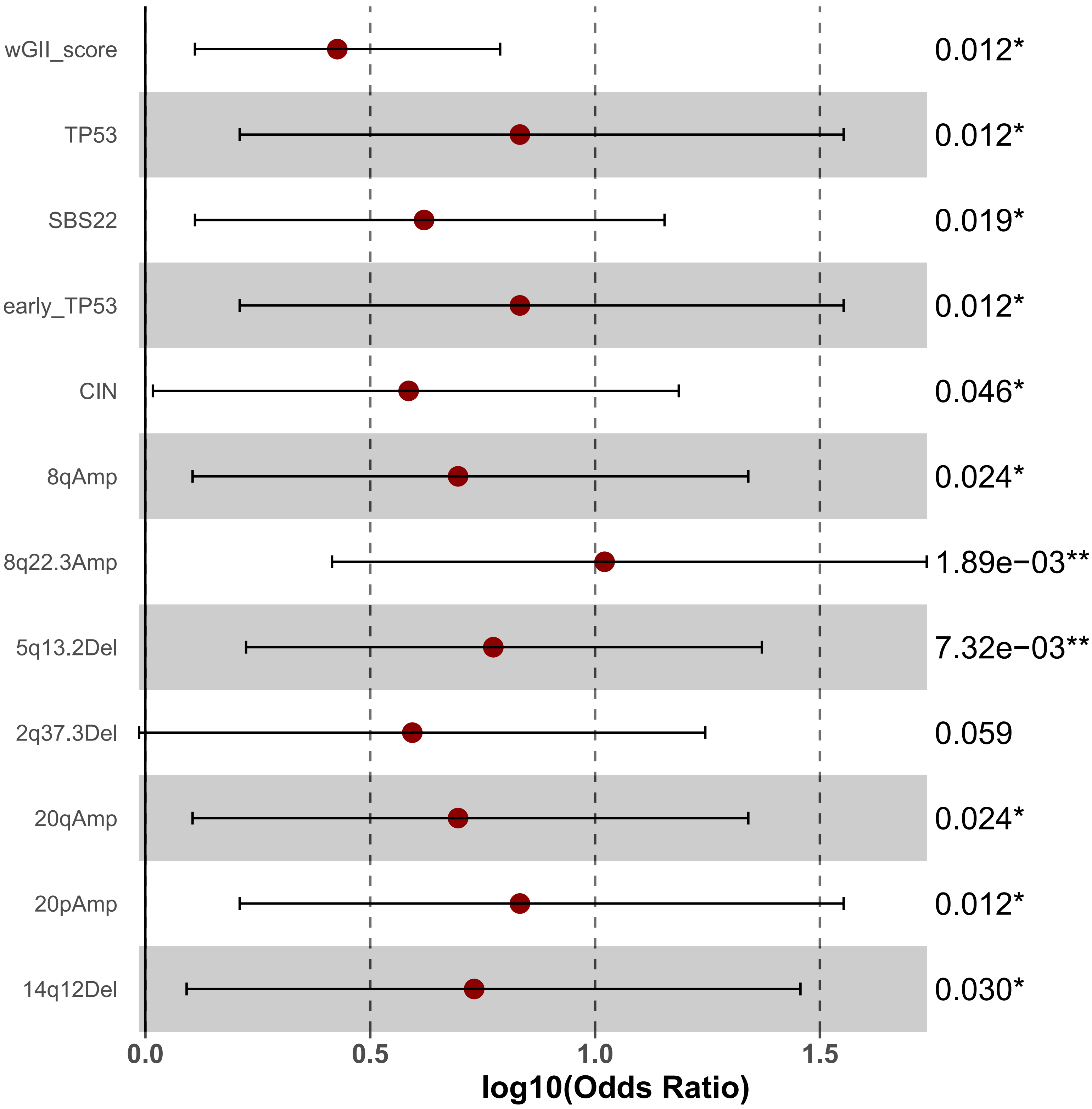

Figure S11: Genomic events associationed with bladder cancer recurrence. (A) Kaplan-Meier analysis in significant genomic events. (B) The forest plot of logistical regression in reference to recurrent group. The corresponding odds ratio and 95% confidence interval (CI) are shown.

Figure S12

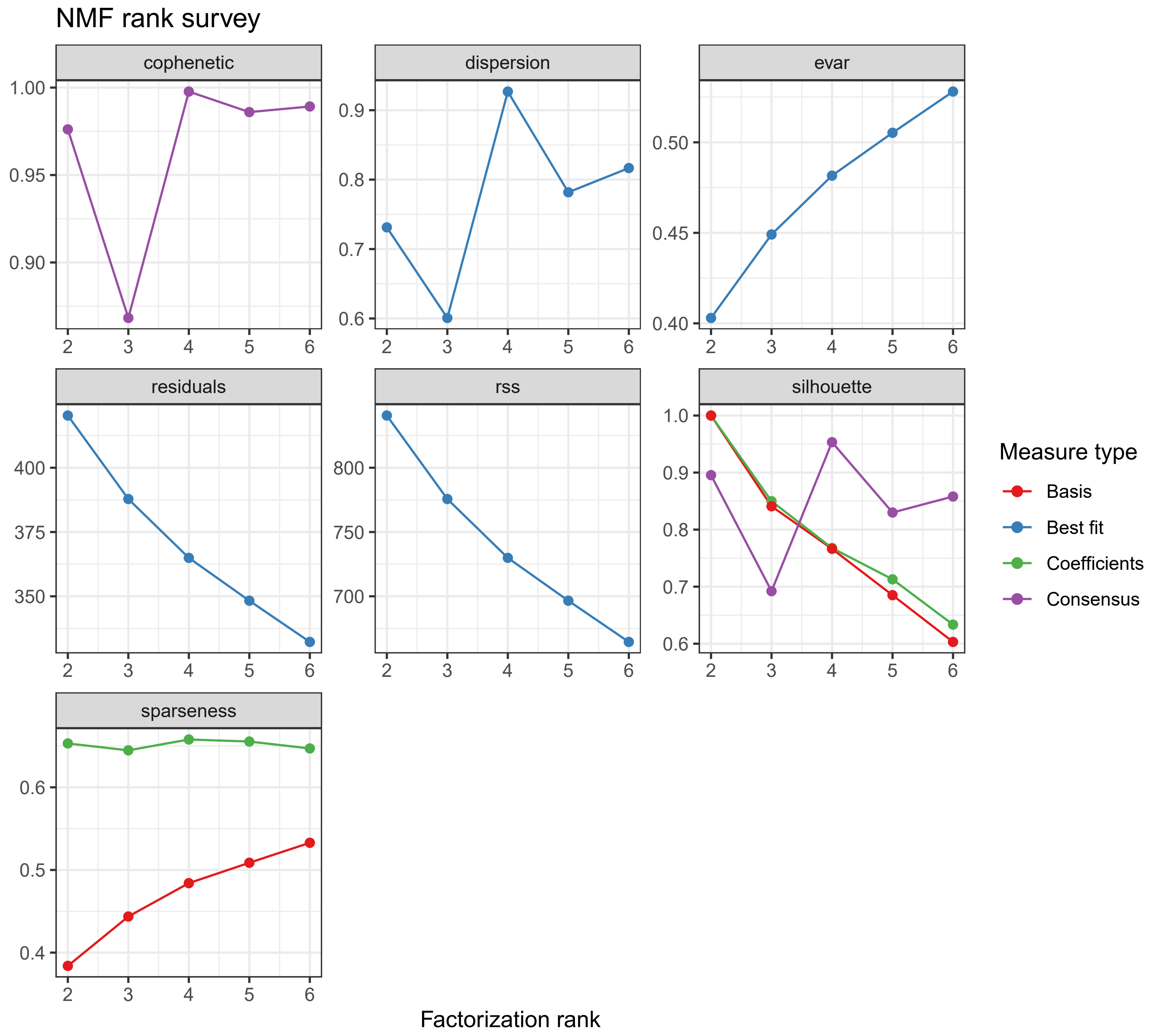

Figure S12: Choosing the best rank of NMF

Figure S13A

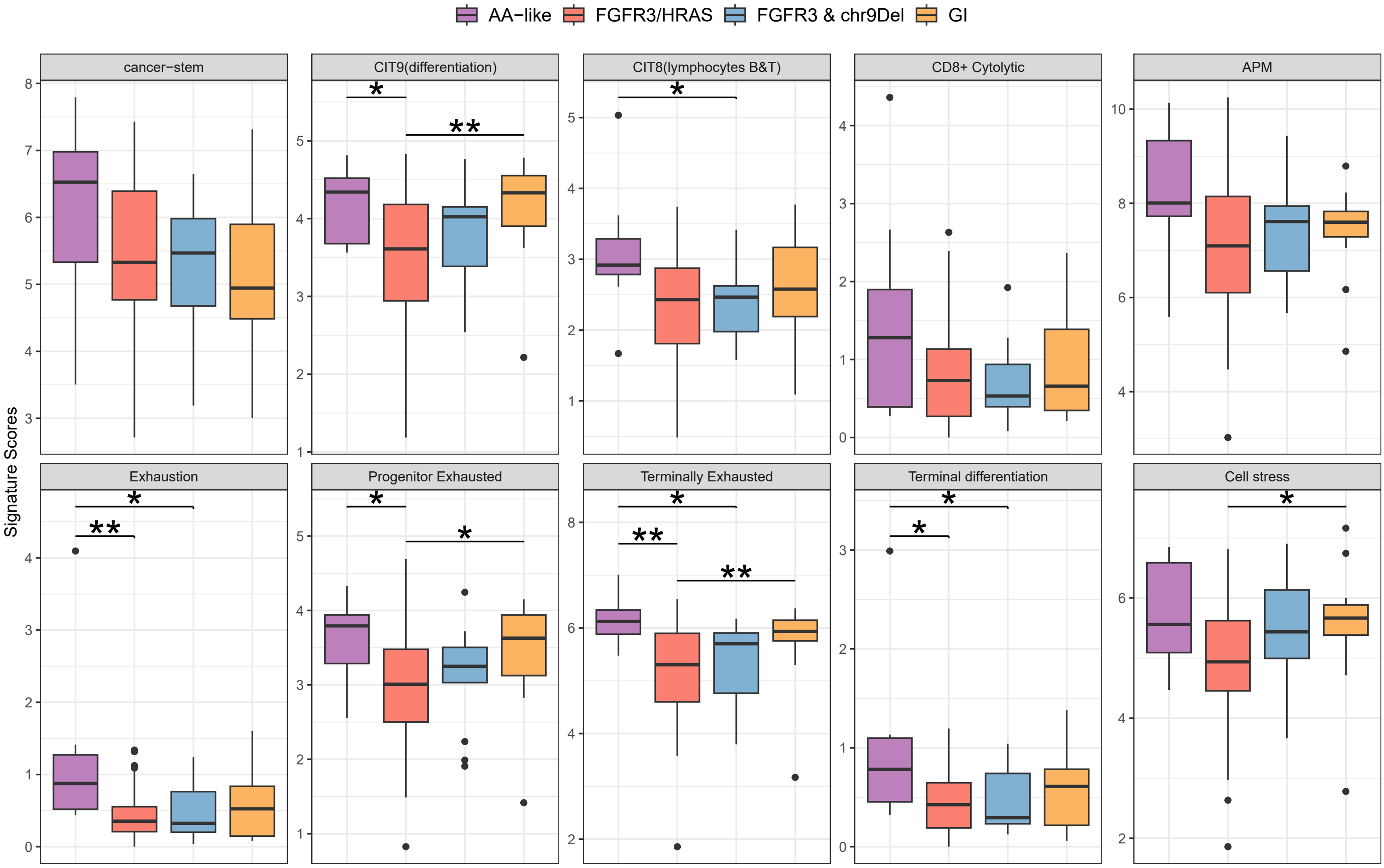

AA−like
FGFR3/HRAS
FGFR3 & chr9Del
GI
cancer−stem
CIT9(differentiation)
CIT8(lymphocytes B&T)
CD8+ Cytolytic
APM
Exhaustion
Progenitor Exhausted
Terminally Exhausted
Terminal differentiation
Cell stress
Signature Scores

Figure S13B

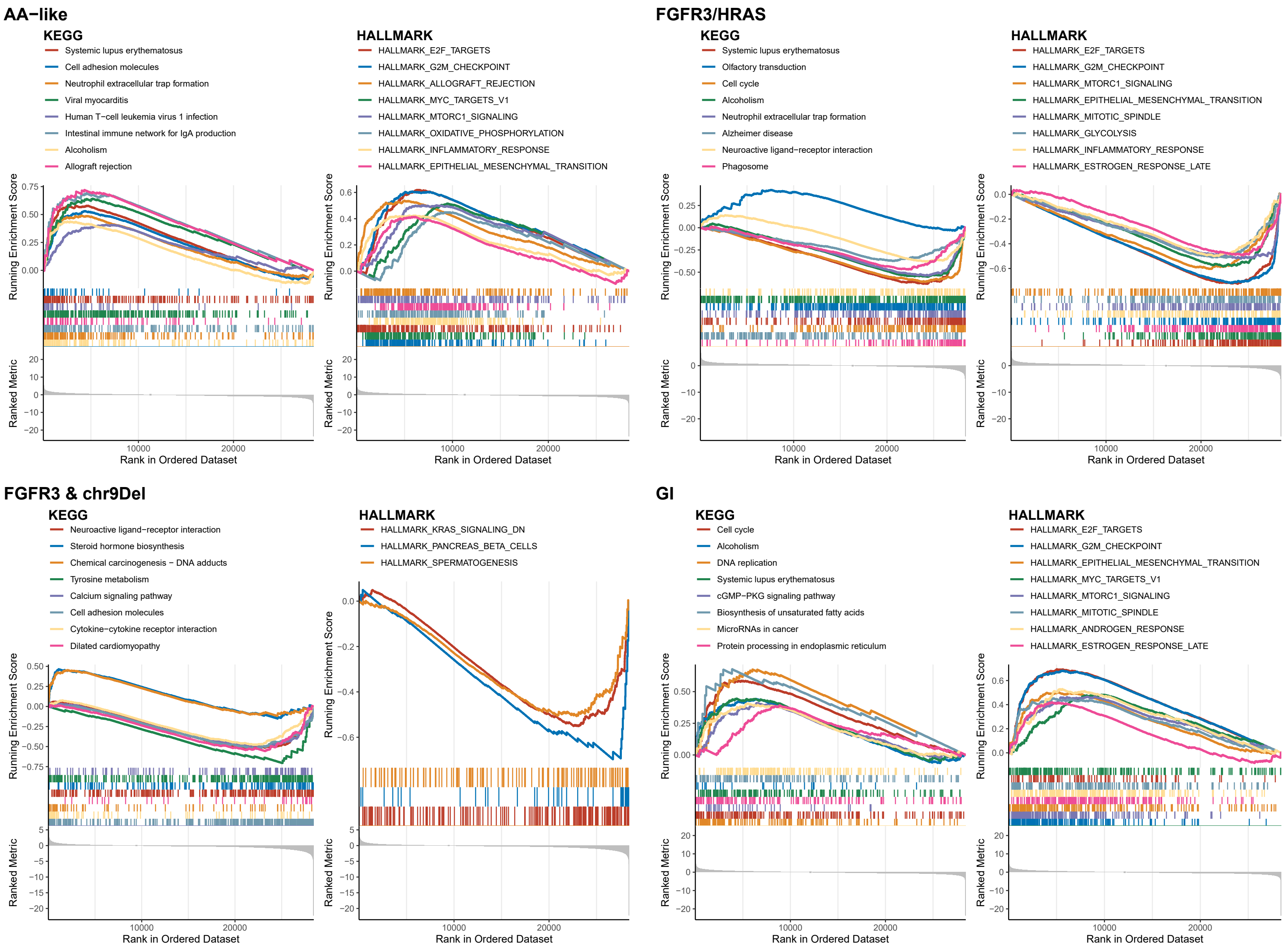
AA−like
KEGG
Systemic lupus erythematosus
Cell adhesion molecules
Neutrophil extracellular trap formation
Viral myocarditis
Human T−cell leukemia virus 1 infection
Intestinal immune network for IgA production
Alcoholism
Allograft rejection
HALLMARK
HALLMARK_E2F_TARGETS
HALLMARK_G2M_CHECKPOINT
HALLMARK_ALLOGRAFT_REJECTION
HALLMARK_MYC_TARGETS_V1
HALLMARK_MTORC1_SIGNALING
HALLMARK_OXIDATIVE_PHOSPHORYLATION
HALLMARK_INFLAMMATORY_RESPONSE
HALLMARK_EPITHELIAL_MESENCHYMAL_TRANSITION
FGFR3/HRAS
KEGG
Systemic lupus erythematosus
Olfactory transduction
Cell cycle
Alcoholism
Neutrophil extracellular trap formation
Alzheimer disease
Neuroactive ligand−receptor interaction
Phagosome
HALLMARK
HALLMARK_E2F_TARGETS
HALLMARK_G2M_CHECKPOINT
HALLMARK_MTORC1_SIGNALING
HALLMARK_EPITHELIAL_MESENCHYMAL_TRANSITION
HALLMARK_MITOTIC_SPINDLE
HALLMARK_GLYCOLYSIS
HALLMARK_INFLAMMATORY_RESPONSE
HALLMARK_ESTROGEN_RESPONSE_LATE
FGFR3 & chr9Del
KEGG
Neuroactive ligand−receptor interaction
Steroid hormone biosynthesis
Chemical carcinogenesis − DNA adducts
Tyrosine metabolism
Calcium signaling pathway
Cell adhesion molecules
Cytokine−cytokine receptor interaction
Dilated cardiomyopathy
HALLMARK
HALLMARK_KRAS_SIGNALING_DN
HALLMARK_PANCREAS_BETA_CELLS
HALLMARK_SPERMATOGENESIS
GI
KEGG
Cell cycle
Alcoholism
DNA replication
Systemic lupus erythematosus
cGMP−PKG signaling pathway
Biosynthesis of unsaturated fatty acids
MicroRNAs in cancer
Protein processing in endoplasmic reticulum
HALLMARK
HALLMARK_E2F_TARGETS
HALLMARK_G2M_CHECKPOINT
HALLMARK_EPITHELIAL_MESENCHYMAL_TRANSITION
HALLMARK_MYC_TARGETS_V1
HALLMARK_MTORC1_SIGNALING
HALLMARK_MITOTIC_SPINDLE
HALLMARK_ANDROGEN_RESPONSE
HALLMARK_ESTROGEN_RESPONSE_LATE
Running Enrichment Score
Ranked Metric
Rank in Ordered Dataset
10000
20000

Figure S13C

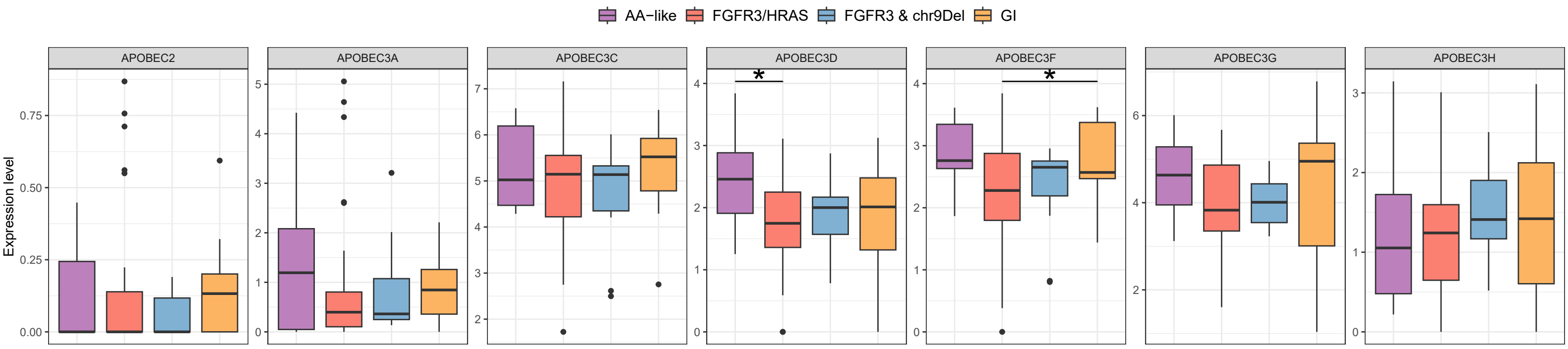

Figure S13D

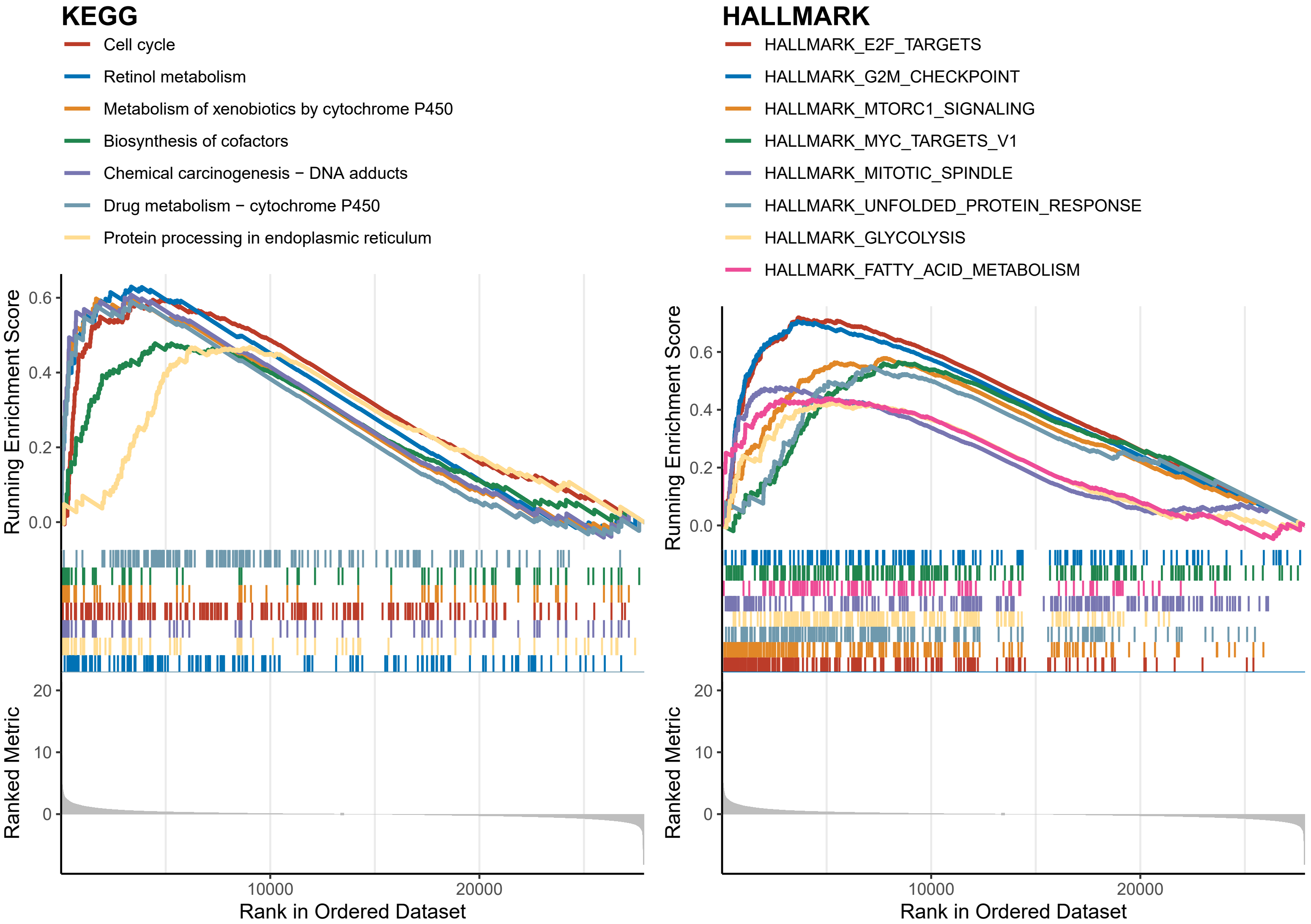

Figure S13: Characteristics of the genomic subtypes. (A) Gene expression signature scores for gene sets were compared. (B) GSEA was employed to reveal the function of four genomic subtypes. (C) Expression levels of APOBEC family members were compared between genomic subypes. (D) GSEA was employed to reveal the function of APOBEC mutation load in FGFR3/HRAS, FGFR3 & ch9Del and GI subtypes.

Figure S14A

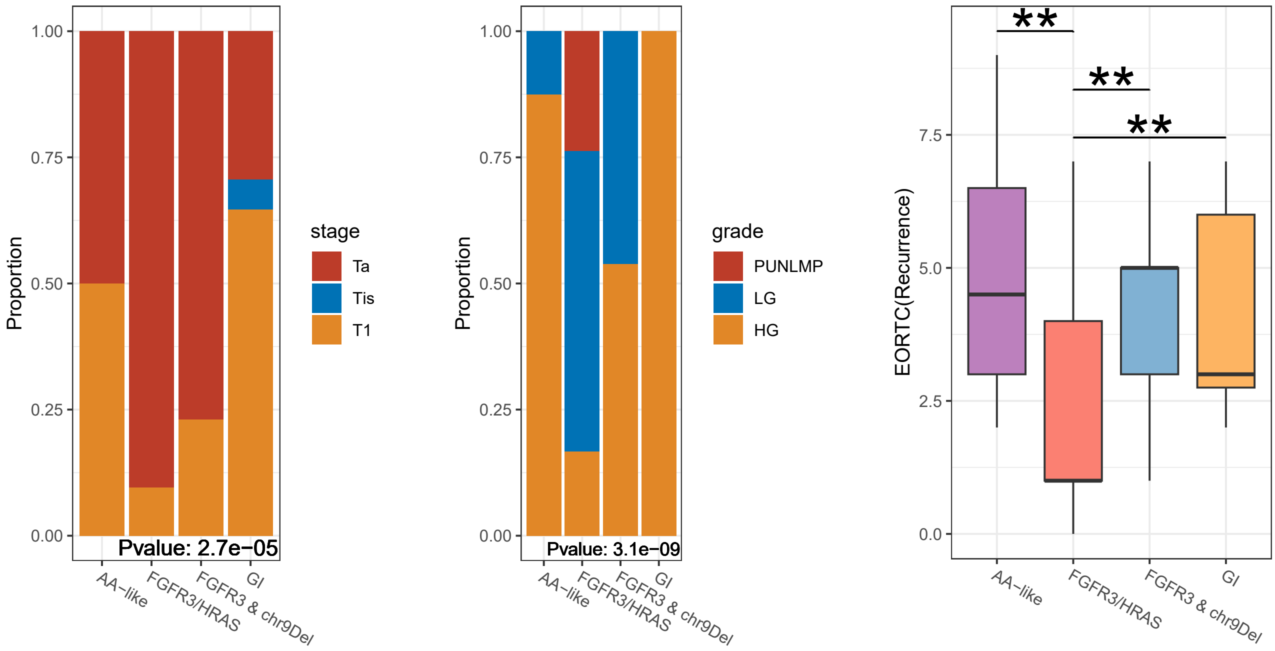

Figure S14B

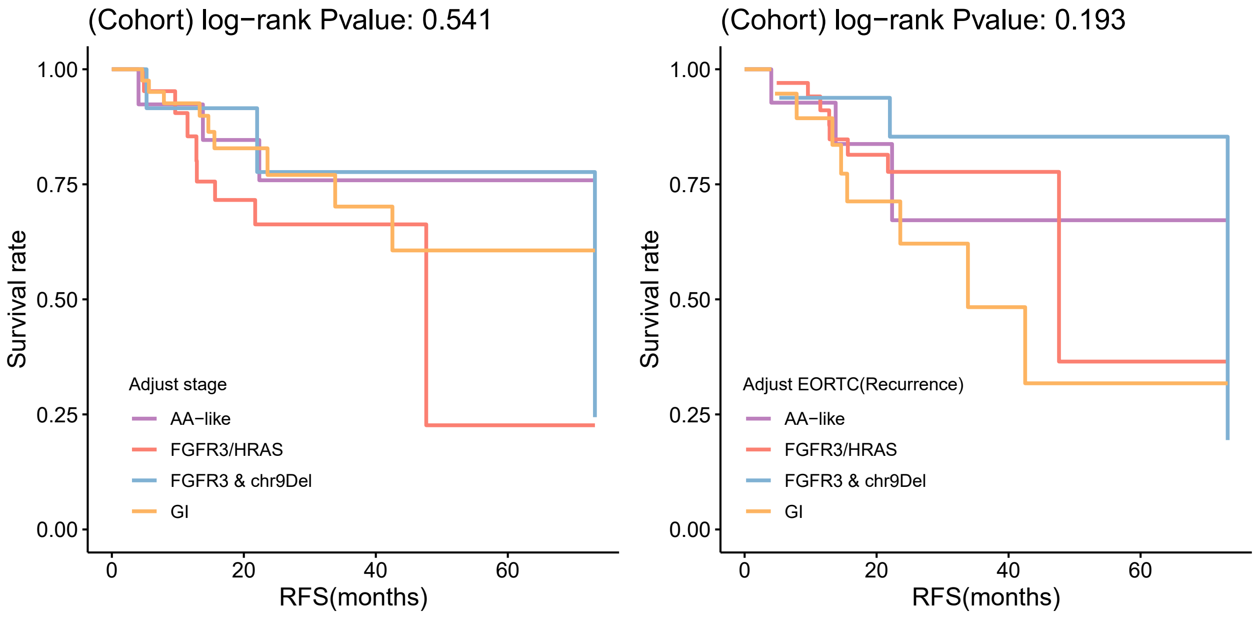

Figure S14C

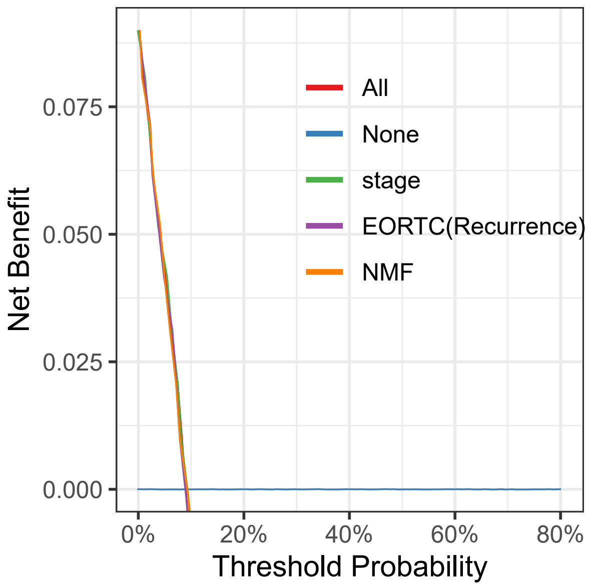

Figure S14D

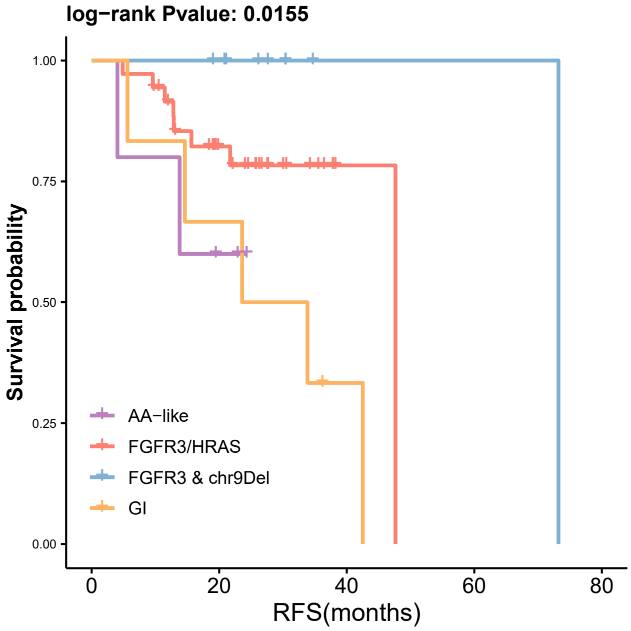

Figure S14F

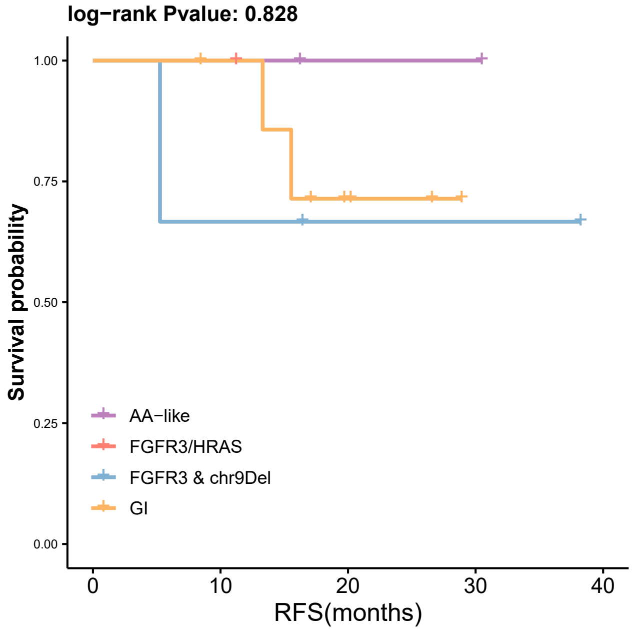

Figure S14E

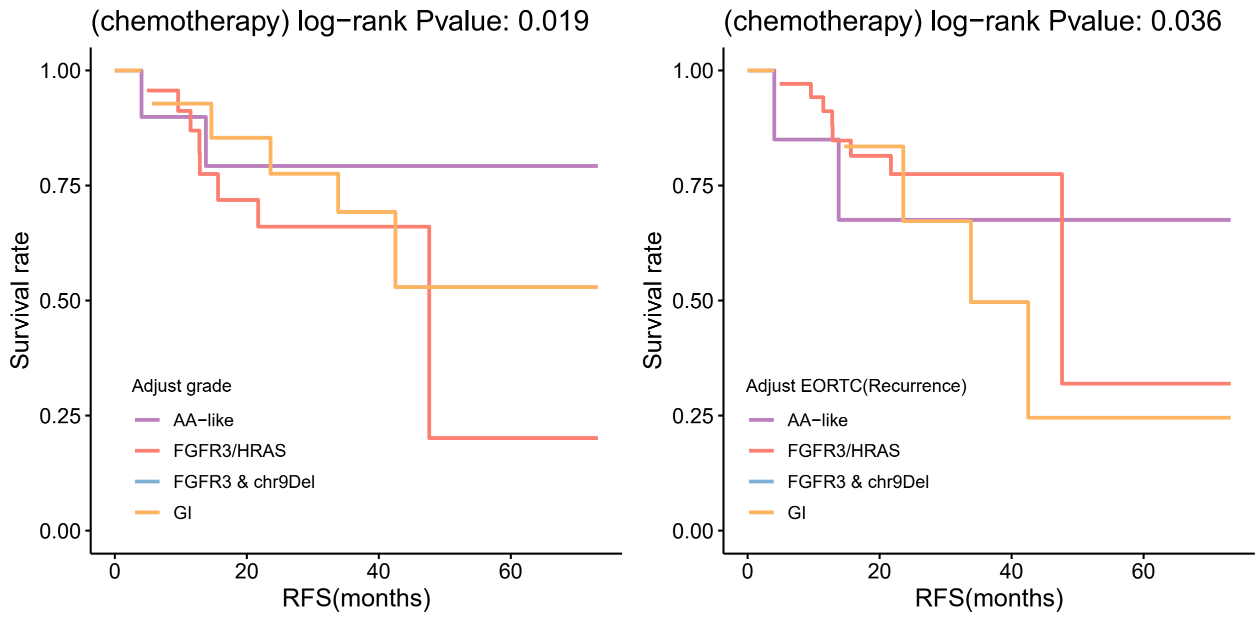

Figure S14: Clinical implications of the genomic subtypes (A) Associations between genomic subtypes and clinical characteristics. (B) Kaplan-Meier plot in all samples. (C) Decision curve analysis. Note: Random noise is added to the curves for "stage", "EORTC(Recurrence)", "NMF", and "all" to avoid overlapping. (D) Kaplan-Meier plot in intravesical chemotherapy samples without adjusting for clinical characteristics. (E) Kaplan-Meier plot in intravesical chemotherapy samples, adjusted for clinical characteristics. (F) Kaplan-Meier plot in BCG-treated samples without adjusting for clinical characteristics.
