# Supplementary material for: Genomic subtypes of non-muscle-invasive bladder cancer: guiding immunotherapy decision-making for patients exposed to aristolochic acid
Source: Mol Med. 2025 Apr 17;31:140. doi: 10.1186/s10020-025-01199-1 (PMC12004710; doi:10.1186/s10020-025-01199-1)
Supplement: Supplementary file 2 — Additional file 2 [file 10020_2025_1199_MOESM2_ESM.docx]

**Supplementary Experimental methods**

**Whole Exome Sequencing (WES)**

WES covering 60.3 megabases of 25946 genes was performed. The mean depth of coverage for targeted regions was 111-fold (ranging from 72-fold to 172-fold) in blood samples and 136-fold (ranging from 86-fold to 215-fold) in tumor samples. The quality of isolated genomic DNA was verified by using these two methods in combination: 1. DNA degradation and contamination were monitored on 1% agarose gels; 2. DNA concentration was measured by Qubit® DNA Assay Kit in Qubit® 2.0 Flurometer (Invitrogen, USA). A total amount of 0.6 μg genomic DNA per sample was used as input material for the DNA sample preparation. Sequencing libraries were generated using Agilent SureSelect Human All Exon V6 kit (Agilent Technologies, CA, USA) following manufacturer’s recommendations and index codes were added to each sample Briefly, fragmentation was carried out by hydrodynamic shearing system (Covaris, Massachusetts, USA) to generate 180-280 bp fragments. Remaining overhangs were converted into blunt ends via exonuclease/polymerase activities. After adenylation of 3’ ends of DNA fragments, adapter oligonucleotides were ligated. DNA fragments with ligated adapter molecules on both ends were selectively enriched in a PCR reaction. After PCR reaction, libraries hybridize with liquid phase with biotin labeled probe, then use magnetic beads with streptomycin to capture the exons of genes. Captured libraries were enriched in a PCR reaction to add index tags to prepare for sequencing. Products were purified using AMPure XP system (Beckman Coulter, Beverly, USA) and quantified using the Agilent high sensitivity DNA assay on the Agilent Bioanalyzer 2100 system. The clustering of the index-coded samples was performed on a cBot Cluster Generation System using Hiseq PE Cluster Kit (Illumina) according to the manufacturer’s instructions. After cluster generation, the DNA libraries were sequenced on Illumina Hiseq platform and 150 bp paired-end reads were generated.

**Variant calling**

The GATK (v4.4.0.0) best practices workflow(1,2) (https://gatk.broadinstitute.org) was used for variant discovery analysis. Initial quality control of raw DNA paired-end reads was performed using FastQC (v.0.11.9, <https://www.bioinformatics.babraham.ac.uk/projects/fastqc/>). Subsequently, fastp(3) (v.0.23.2, flags: -q 20; -u 40; --detect_adaper_for_pe; --correction; --overrepresentation_analysis) was used to remove adapter sequences and trim unqualified reads. Trimmed reads were aligned to the hg38 genome assembly using BWA-MEM(4) (v.0.7.17) and were then sorted using samtools(5) (1.13). To ensure accurate variant calling and reliable mutation timing, specific criteria were applied to determine true positive variants. In tumor samples, a variant was considered a true positive if the variant allele frequency (VAF) was greater than 5% and the number of reads supporting the variant was equal to or greater than 5. In germline data, the number of reads supporting the variant had to be less than 5, and the VAF had to be less than or equal to 1%. Furthermore, to ensure sufficient sequencing coverage, the sequencing depth in each region had to be ≥ 30 in tumor samples and ≥ 10 in normal samples (6–8). Finally, Variants were annotated using Ensembl Variant Effect Predictor (v109)(9).

**Copy number analysis**

The alleleCount tool (http://cancerit.github.io/alleleCount/) was initially utilized to obtain allele counts at genomic SNP loci (based on the 1000 genomes project(10)) from a pair of tumor/normal BAM files. These allele counts were then converted into logR (log ratio) and BAF (B-allele frequency) values using ASCAT (v3.1.2)(11), which is a software used for inferring allele-specific copy number information. The logR and BAF values for each sample were further processed with ASCAT using default parameters, with the exception of setting the "gamma" parameter to 1 for sequencing data. This processing allowed for the generation of segmented allele-specific copy number data, as well as estimates of cellularity and ploidy for all samples. In the copy number analysis, floating point copy number values were used(7).

**RNA sequencing**

A total amount of 1 µg RNA per sample was used as input material for the RNA sample preparations. Sequencing libraries were generated using NEBNext® UltraTM RNA Library Prep Kit for Illumina® (NEB, USA) following manufacturer’s recommendations and index codes were added to attribute sequences to each sample. Briefly, mRNA was purified from total RNA using poly-T oligo-attached magnetic beads. Fragmentation was carried out using divalent cations under elevated temperature in NEBNext First Strand Synthesis Reaction Buffer(5X). First strand cDNA was synthesized using random hexamer primer and M-MuLV Reverse Transcriptase (RNase H). Second strand cDNA synthesis was subsequently performed using DNA Polymerase I and RNase H. Remaining overhangs were converted into blunt ends via exonuclease/polymerase activities. After adenylation of 3’ ends of DNA fragments, NEBNext Adaptor with hairpin loop structure were ligated to prepare for hybridization. In order to select cDNA fragments of preferentially 250~300 bp in length, the library fragments were purified with AMPure XP system (Beckman Coulter, Beverly, USA). Then 3 µl USER Enzyme (NEB, USA) was used with size-selected, adaptor-ligated cDNA at 37°C for 15 min followed by 5 min at 95 °C before PCR. Then PCR was performed with Phusion High-Fidelity DNA polymerase, Universal PCR primers and Index (X) Primer. At last, PCR products were purified (AMPure XP system) and library quality was assessed on the Agilent Bioanalyzer 2100 system. The clustering of the index-coded samples was performed on a cBot Cluster Generation System using TruSeq PE Cluster Kit v3-cBot-HS (Illumia) according to the manufacturer’s instructions. After cluster generation, the library preparations were sequenced on an Illumina Novaseq platform and 150 bp paired-end reads were generated.

**Gene expression quantification**

The raw RNA paired-end reads underwent initial quality control using FastQC (v.0.11.9, https://www.bioinformatics.babraham.ac.uk/projects/fastqc/). Subsequently, the fastp(3) tool (v.0.23.2, with flags: -q 20; -u 40; --detect_adapter_for_pe; --correction; --overrepresentation_analysis; --dedup; --dup_calc_accuracy 6) was employed to remove adapter sequences and trim unqualified reads. Transcript quantification was performed using salmon (v.0.14.2)(12), and the results were then summarized at the gene level using tximport(13).

**Gene expression signature scores**

The raw gene expression signature score was defined as a mean of log2(TPM + 1) for basal markers(14) (CD44, CDH3, KRT1, KRT14, KRT16, KRT5, KRT6A, KRT6B, KRT6C), luminal markers(14) (CYP2J2, ERBB2, ERBB3, FGFR3, FOXA1, GATA3, GPX2, KRT18, KRT19, KRT20, KRT7, KRT8, PPARG, XBP1, UPK1A, UPK2), p53-like markers(14) (ACTG2, CNN1, MYH11, MFAP4, PGM5, FLNC, ACTC1, DES, PCP4), squamous-differentiation markers(15) (DSC1, DSC2, DSC3, DSG1, DSG2, DSG3, S100A7, S100A8), neuroendocrine markers(15) (CHGA, CHGB, SCG2, ENO2, SYP, NCAM1), extracellular matrix (ECM) and smooth muscle markers, cell-cycle genes(16), cancer-stem cell markers(17) (CD44, KRT5, RPSA, ALDH1A1I), a set of markers known to be associated with epithelial-mesenchymal transition (EMT)(15) (ZEB1, ZEB2, VIM, SNAIL, TWIST1, FOXC2, CDH2), claudin-low markers(14) (CLDN3, CLDN7, CLDN4, CDH1, VIM, SNAI2, TWIST1, ZEB1, ZEB2), Cartes d’Identité des Tumeurs (CIT) gene sets(18) and BCG response markers(19) (MAEA, SEC24C, ZC3HC1, UBR4, AP1G1, UBE2I, HLA-A, RNPS1, PTPRM, BTG2, PITRM1, POLR2A, STOML2, RNF20, XRCC5, MSRB1, TAX1BP3, LGMN). The CIT sets included tumor cell component 9; stromal components 3, 8, and 12; and components 5 and 14, which could not be attributed to either tumor or stromal cells.

**Functional enrichment analysis**

Functional enrichment analysis, including overrepresentation analysis (ORA) and gene set enrichment analysis (GSEA), was conducted using the clusterProfiler(20) package. In this analysis, only hallmark gene sets from the Molecular Signatures Database (MsigDb)(21) and the cancer pathways with a minimum of 5 genes and a maximum of 500 genes were considered. An adjusted P value of < 0.05 was regarded as significant.

**References**

1. DePristo MA, Banks E, Poplin R, Garimella KV, Maguire JR, Hartl C, et al. A framework for variation discovery and genotyping using next-generation DNA sequencing data. Nat Genet. 2011 May;43(5):491–8.

2. McKenna A, Hanna M, Banks E, Sivachenko A, Cibulskis K, Kernytsky A, et al. The Genome Analysis Toolkit: A MapReduce framework for analyzing next-generation DNA sequencing data. Genome Res. 2010 Jan 9;20(9):1297–303.

3. Chen S, Zhou Y, Chen Y, Gu J. fastp: an ultra-fast all-in-one FASTQ preprocessor. Bioinformatics. 2018 Sep 1;34(17):i884–90.

4. Li H, Durbin R. Fast and accurate short read alignment with Burrows–Wheeler transform. Bioinformatics. 2009 Jul 15;25(14):1754–60.

5. Li H, Handsaker B, Wysoker A, Fennell T, Ruan J, Homer N, et al. The Sequence Alignment/Map format and SAMtools. Bioinformatics. 2009 Aug 15;25(16):2078–9.

6. McGranahan N, Favero F, De Bruin EC, Birkbak NJ, Szallasi Z, Swanton C. Clonal status of actionable driver events and the timing of mutational processes in cancer evolution. Sci Transl Med [Internet]. 2015 Apr 15 [cited 2023 Jun 7];7(283). Available from: https://www.science.org/doi/10.1126/scitranslmed.aaa1408

7. Jamal-Hanjani M, Wilson GA, McGranahan N, Birkbak NJ, Watkins TBK, Veeriah S, et al. Tracking the Evolution of Non–Small-Cell Lung Cancer. New Engl J Med. 2017 Jun;376(22):2109–21.

8. McGranahan N, Furness AJS, Rosenthal R, Ramskov S, Lyngaa R, Saini SK, et al. Clonal neoantigens elicit T cell immunoreactivity and sensitivity to immune checkpoint blockade. Science. 2016 Mar 25;351(6280):1463–9.

9. McLaren W, Gil L, Hunt SE, Riat HS, Ritchie GRS, Thormann A, et al. The Ensembl Variant Effect Predictor. Genome Biol. 2016 Jun 6;17(1):122.

10. Auton A, Abecasis GR, Altshuler DM, Durbin RM, Abecasis GR, Bentley DR, et al. A global reference for human genetic variation. Nature. 2015 Oct;526(7571):68–74.

11. Allele-specific copy number analysis of tumors | PNAS. [cited 2021 May 12]; Available from: https://www.pnas.org/content/107/39/16910

12. Patro R, Duggal G, Love MI, Irizarry RA, Kingsford C. Salmon provides fast and bias-aware quantification of transcript expression. Nat Methods. 2017 Apr;14(4):417–9.

13. Soneson C, Love MI, Robinson MD. Differential analyses for RNA-seq: transcript-level estimates improve gene-level inferences [Internet]. F1000Research; 2016 [cited 2023 Jul 12]. Available from: https://f1000research.com/articles/4-1521

14. Dadhania V, Zhang M, Zhang L, Bondaruk J, Majewski T, Siefker-Radtke A, et al. Meta-Analysis of the Luminal and Basal Subtypes of Bladder Cancer and the Identification of Signature Immunohistochemical Markers for Clinical Use. Ebiomedicine. 2016 Oct 1;12:105–17.

15. Robertson AG, Kim J, Al-Ahmadie H, Bellmunt J, Guo G, Cherniack AD, et al. Comprehensive Molecular Characterization of Muscle-Invasive Bladder Cancer. Cell. 2017 Oct;171(3):540-556.e25.

16. Cuzick J, Swanson GP, Fisher G, Brothman AR, Berney DM, Reid JE, et al. Prognostic value of an RNA expression signature derived from cell cycle proliferation genes in patients with prostate cancer: a retrospective study. The Lancet Oncology. 2011 Mar 1;12(3):245–55.

17. Chan KS, Volkmer JP, Weissman I. Cancer stem cells in bladder cancer: a revisited and evolving concept. Curr Opin Urol. 2010 Sep;20(5):393–7.

18. Biton A, Bernard-Pierrot I, Lou Y, Krucker C, Chapeaublanc E, Rubio-Pérez C, et al. Independent Component Analysis Uncovers the Landscape of the Bladder Tumor Transcriptome and Reveals Insights into Luminal and Basal Subtypes. Cell Rep. 2014 Nov 20;9(4):1235–45.

19. Kim YJ, Ha YS, Kim SK, Yoon HY, Lym MS, Kim MJ, et al. Gene Signatures for the Prediction of Response to Bacillus Calmette-Guérin Immunotherapy in Primary pT1 Bladder Cancers. Clin Cancer Res. 2010 Apr 1;16(7):2131–7.

20. Wu T, Hu E, Xu S, Chen M, Guo P, Dai Z, et al. clusterProfiler 4.0: A universal enrichment tool for interpreting omics data. The Innovation. 2021 Aug 28;2(3):100141.

21. Liberzon A, Birger C, Thorvaldsdóttir H, Ghandi M, Mesirov JP, Tamayo P. The Molecular Signatures Database (MSigDB) hallmark gene set collection. Cell Syst. 2015 Dec 23;1(6):417–25.
